# pH-Responsive, Thermoset Polymer Coatings for Active Protection against Aluminum Corrosion

**DOI:** 10.1021/acsami.3c14752

**Published:** 2024-03-01

**Authors:** Joseph Watson, Victoria Balmforth, Elaine Gray, Matthew G. Unthank

**Affiliations:** † Northumbria University, Newcastle upon Tyne NE1 8ST, U.K.; ‡ AkzoNobel, Polymer Development Group, Stoneygate Lane, Felling, Tyne & Wear NE10 0JY, U.K.

**Keywords:** coating, polymers, polymer coatings, material interfaces, corrosion protection, filiform corrosion, responsive polymers

## Abstract

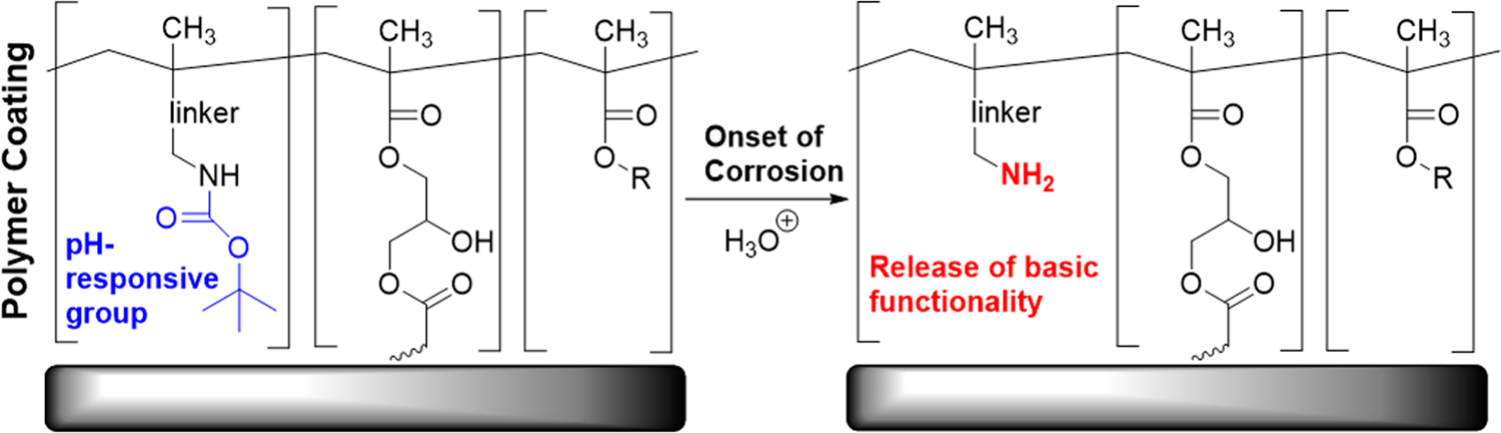

This paper describes the synthesis and use of multifunctional methacrylic monomers, which contain basic (amine) functional groups, including an example in which an acid-labile *tert*-butylcarbamate-protected glycine is used to form a novel methacrylic monomer. The “protected” amino acid-derived functional monomer (BOC-Gly-MA) is copolymerized with an epoxide functional methacrylic monomer (GMA), to deliver novel multifunctional polymers, which are processed into powder coatings and used to study filiform corrosion at the surface of an aluminum substrate. The BOC-Gly-MA-containing copolymers were shown to improve a coating’s anticorrosion performance, presenting the lowest average filiform corrosion (FFC) track length, total FFC number, and total corroded surface area (CSA) of the coatings investigated. Further to this, a mode of action for the role of BOC-Gly functional polymers in corrosion protection is proposed, supported by both solution and polymer–aluminum interface studies, delivering new insights into the mode of action of pH-responsive polymer coatings.

## Introduction

Organic polymer coatings provide corrosion protection to metal substrates and are widely used across a range of industries, from architectural, marine, and automotive.^1,2^ Powder coatings are extensively used in the automotive industry due to their ability to effectively and efficiently coat metallic substrates via electrostatic application methods. In this process, charged powder coating particles (consisting of polymers, cross-linkers, and additives) are attracted to the electrically earthed substrate, producing a thin uniform coating across the entirety of the metal surface.^3−5^ High-optical clarity powder coatings for high aesthetic demand applications, such as the protection of aluminum alloy wheels, are typically based on epoxide functional acrylic copolymers, cross-linked with flexible C8–C12 diacids such as sebacic or dodecanoic acid. Such coatings deliver a combination of excellent mechanical properties, chemical resistance, and optical clarity.^3,6−10^ Polymer coatings act as a physical barrier protecting the metal surface from corrosion; however, this passive protection is only effective as long as the coating remains intact and the entire wheel (including edges) is coated with a protective layer of polymer.^11−13^

A major performance challenge of polymer-coated aluminum is the material’s susceptibility to filiform corrosion (FFC).^14^ FFC initiates at areas of coating damage, defects, or low film thickness, which allows corrosive media (particularly aqueous chlorides and acids) to reach the metal surface.^15^ Water, oxygen, and ions permeate the coating and the FFC tail, fueling the redox process at the filiform head, thus causing FFC propagation under the coating, increasing the degree of damage and loss of attractive aesthetic appearance.^16^ Coatings designed solely for barrier protection only remain effective in corrosion prevention when undamaged and adhered to a metallic surface. Due to this limitation, chemically ‘active corrosion protection’ additives are often dispersed within the organic coatings to improve their protective abilities.^17^ The main routes of achieving active corrosion protection are through incorporation of reactive species into the coatings such as inorganic fillers, for instance, CeCl_3_, Li_2_CO_3_, or MoO_2_^–4^ (and historically chromates),^17^ or alternatively through dispersion of low-solubility organic compounds including 2-mercaptobenzothiazole, imidazole derivatives, or benzotriazoles, dispersed either within the coating or contained within microcapsules.^18−21^

Previous research has measured the pH of the filiform head to be within the range of pH 1.0–2.5,^22,23^ caused by high concentrations of Al^3+^ ions in solution. As the aqueous acidic conditions within a filiform corrosion cell are well described, polymers (and subsequent polymeric coatings) could potentially be designed to act upon these environmental triggers. Research has studied the use of semisoluble lithium salts (Li_2_CO_3_ and Li_2_C_2_O_4_), which can inhibit corrosion through increasing pH and promoting the formation of a protective layer at the damaged coating area.^24−32^ Upon coating damage and exposure to a corrosive environment, lithium salts were reported to leach from the coating to the site of coating damage where they were able to form a passivating layer, protecting the coating surface from further corrosion propagation. However, the dispersion of lithium salts in the otherwise transparent organic coatings resulted in an opaque finish, and as such, this approach is not viable for high-optical clarity powder coatings, such as those required for the protection of aluminum alloy wheels.

**Figure 1:**
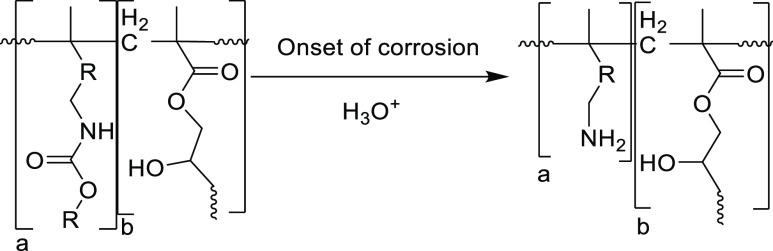
Proposed mode of action for pH-activated protecting group removal, post-polymerization.

In the research described here, we propose that the inclusion of selected chemical functionalities within the polymer backbone itself may be a route to minimization of FFC (through increasing pH), while also maintaining optical clarity. It was hypothesized that a polymer tethered with amine functionalities may be suitable for this process, as they would buffer the low (acidic) pH at the propagating filiform head, increasing pH. However, the inclusion of an amine functionality in epoxy-based thermosetting polymer coatings for aluminum protection presents significant technical challenges. Amines are well known to be (a) highly reactive nucleophiles that undergo (often multiple) addition reactions with epoxide groups, also used in thermosetting powder coatings,^33,34^ and (b) prone to discoloration in coatings (via *N*-oxidation).^35^ One solution to this issue would be through the use of amine-protecting groups, where deprotection (and concomitant liberation of the basic amine group) occurs under the conditions of filiform corrosion (i.e., pH ∼ 1.0–2.5). The *tert*-butylcarbamate (BOC) protecting group is widely used in the fine chemical and pharmaceutical industries as an acid-labile protecting group for amines^36−41^ but to the best of our knowledge has never been studied in coatings for pH-responsive corrosion prevention (Figure 1). The use of this functional group may open opportunities to create multifunctional, pH-responsive polymers capable of both forming thermoset coatings and preventing propagation of FFC on aluminum substrates.

## Experimental Section

### Materials

All materials were purchased from commercial vendors listed in the Supporting Information and used as received. The commercial control polymer (COM) was supplied by the industrial sponsor AkzoNobel and used as received.

### Nuclear Magnetic Resonance (NMR)

NMR analyses were acquired at 25 °C using a JEOL ECS400 Delta spectrometer at frequencies of 399.78 MHz for ^1^H NMR and 100.53 MHz for ^13^C NMR. All chemical shifts are quoted as parts per million (ppm) relative to tetramethylsilane (TMS, δ = 0 ppm) as an internal standard in either deuterated chloroform (CDCl_3_) or deuterated dimethyl sulfoxide (DMSO-*d*_6_). ^13^C NMR assignment was confirmed by DEPT analysis. The spectral data are recorded as a chemical shift (δ), relative integral, multiplicity (s = singlet, br = broad, d = doublet, t = triplet, q = quartet, quin = quintet, sext = sextet, dd = doublet of doublets, m = multiplet), and coupling constant (*J* = Hz).

### Fourier Transform Infrared Spectroscopy (FTIR)

Infrared spectroscopy was performed on a Bruker α-platinum-ATR or a PerkinElmer Spotlight 150i FT-IR microscope, with an aperture of 100 μm × 100 μm, and the output data were analyzed in OPUS or Spectrum 10 software, respectively. Absorption maxima are expressed in wavenumbers (cm^–1^).

### Gel Permeation Chromatography (GPC)

Samples were dissolved in THF (2 mg/mL) and filtered through 0.2 μm nylon filters. Samples were analyzed using an Agilent 1260 infinity II system equipped with a refractive index and viscometry detector fitted with PLgel MiniMIX-E and PLgel MiniMIX-D columns in sequence, using a THF mobile phase and a flow rate of 0.6 mL/min. Analysis was performed against a calibration curve of polystyrene standards (EasiVial PS-M supplied by Agilent).

### Thermogravimetric Analysis (TGA)

TGA analysis was conducted using a PerkinElmer Pyris 6 TGA thermal analyzer, and results were interpreted in Pyris software (version 11.1.1.0492). Polymer samples of 16–20 mg weight were heated in ceramic crucibles from 30 to 650 °C at a constant rate of 20 °C/min, under a flow of N_2_ (40 mL/min). The residual mass was recorded as a percentage weight of the original mass that remained at 600 °C (within the range where mass remained constant after the degradation of the organic material).

### Tensile Testing

Thin free film polymer samples for mechanical testing were laser-cut to 90 mm × 10 mm using a Glowforge Basic laser cutter equipped with a 40 W CO_2_ laser tube. Tensile experiments were conducted on either an Instron 5969 or Instron 3343 with a displacement ramp rate of 10 mm/min using a 1 kN load cell, and samples were held in place with mechanical wedge grips.

### Synthesis of BOC-Gly-MA

To a solution of *N*-(*tert*-butoxycarbonyl)glycine (BOC-Gly, 14.730 g, 84.0 mmol) and K_2_CO_3_ (0.967 g, 7.0 mmol) in THF (20 mL, degassed using N_2_) was added glycidyl methacrylate (GMA, 9.950 g, 70.0 mmol) before heating to 50 °C under stirring and N_2_. After 24 h, the reaction mixture was cooled to room temperature and diluted with ethyl acetate (100 mL). The reaction mixture was washed with a saturated NaHCO_3_ solution (3 × 100 mL), and the organic phase was collected and dried over MgSO_4_. The organic phase was then concentrated under reduced pressure, affording the product as a clear oil as a mixture of isomers (19.98 g, 75%) (85% major 15% minor, as determined by ^1^H NMR and liquid chromatography mass spectrometry (LCMS)). The product was a mixture of BOC-Gly-MA and unreacted GMA, and the monomer mixture was determined by ^1^H NMR and used directly in a radical polymerization reaction of multifunctional polymers. A small sample was purified by column chromatography for analytical purposes/δH (400 MHz; CDCl_3_): 6.13 (s, 1H), 5.61 (s, 1H), 5.03 (s, 1H), 4.31–4.19 (m, 4H), 4.17–4.12 (m, 1H), 3.93 (d, *J* = 5.5 Hz, 2H), 2.73 (s, 1H), 1.94 (s, 3H), 1.44 (s, 9H). δC (400 MHz; CDCl_3_): 170.5 (C=O), 167.4 (C=O), 156.2 (C=O), 135.8 (C=C), 126.5 (C=C), 80.3 (C(CH_3_)), 67.8 (C–O), 66.0 (C–O), 65.1 (C–O), 42.4 ((C=O)CNHR), 28.3 (C(CH_3_)), 18.3 (CH_3_). νmax/cm^–1^ = 3380w (broad), 2975w, 1703s, 1520m, 1367w. MS: (+ESI) ([M + H]) major = *m*/*z* 318.155, minor = *m*/*z* 318.15.

### General Method of Small-Scale Polymer Synthesis

A solution of mixed monomers (based on Table 1) and *tert*-butyl peroxy-2-ethylhexanoate (Trigonox 21S) (high-temperature, HT method) or azobis(isobutyronitrile) (AIBN) (low-temperature, LT method) was prepared and diluted with butyl acetate (HT) or isopropyl acetate (LT) (solvent mass equal to 20% of the total monomer mass). The monomer/initiator mixture was then added to a 100 mL, 3-neck round-bottom flask containing butyl acetate (HT) or isopropyl acetate (LT) at a volume equal to 50% of the total monomer mass, preheated to 125 °C (HT) or 85 °C (LT). The monomer/initiator mixture was added by use of a syringe pump at a rate of 0.5 mL/min while under N_2_ and constant stirring provided by a magnetic stirrer. Once the monomer/initiator mixture addition was complete, the reaction mixture was stirred for a further 1 h at 125–130 °C (HT) or 85–90 °C (LT). The polymer–solvent mixture was then poured into aluminum foil-lined trays and placed into a vacuum oven (−1020 mbar) at 100 °C (HT) or 85 °C (LT) for 3 h to remove the solvent and nonpolymerized monomer.

**Table 1:** Monomer and Initiator Compositions for the Synthesis of Functional Polymers^a^

		weight %						
entry	sample	MMA	GMA	STY	*^i^*BOMA	BOC-Gly-MA	DPA-MA	initiator (mol %)
1	CTL-HT	52	28	4	16	0	0	4T
2	CTL-LT	52	28	4	16	0	0	8A
3	5-BOC-HT	47	28	4	16	5	0	4T
4	5-BOC-LT	47	28	4	16	5	0	8A
5	50-BOC-HT	0	28	0	22	50	0	4T
6	CTL-HT*	52	28	4	16	0	0	4T
7	5-BOC-HT*	47	28	4	16	5	0	4T
8	5-BOC-LT*	47	28	4	16	5	0	8A
9	5-DPA-HT*	47	28	4	16	0	5	4T

a * indicates polymers synthesized at a 1 kilo scale; T and A indicate whether Trigonox 21S (T) or AIBN (A) was used as the radical initiator, respectively. MMA (methyl methacrylate), GMA (glycidyl methacrylate), STY (styrene), *i*BOMA (*iso*-bornyl methacrylate), BOC-Gly-MA (reaction product of *N*-(*tert*-butoxycarbonyl)glycine and GMA), and DPA (diiso-propylethylamino-methacrylate). The mass of butyl acetate present in the monomer/initiator mixture was equal to 20% of the total monomer mass. The mass of solvent initially present in the reaction vessel prior to infusion was equal to 50% of the total monomer mass.

### General Method of Large-Scale Polymer Synthesis

A solution of mixed monomers (based on Table 1) and Trigonox 21S (HT) or AIBN (LT) was prepared and diluted with butyl acetate (HT and LT) (solvent mass equal to 20% of the total monomer mass). The monomer/initiator mixture was then added to a preheated 2 L flange-neck flask containing butyl acetate (at a volume equal to 50% of the monomer mass) and preheated to 125 °C (HT) or 85 °C (LT). The monomer/initiator mixture was added via use of a peristaltic pump at a rate of 7.4 mL/min over 3 h while under N_2_ and constant stirring provided by an overhead stirrer. Once the monomer/initiator mixture addition was complete, the reaction mixture was stirred for a further 1 h at 125–130 °C (HT) or 85–90 °C (LT). A solution of Trigonox 21S (HT) or AIBN (LT) equal to 10% mass of initial initiator weight was then added to the reaction as a single charge, while stirring at a temperature of 125–130 °C (HT) or 85–90 °C (LT) was maintained for a further 45 min. The reaction was then maintained at 100 °C, and a distillation was performed to remove most of the reaction solvent. After 40 min of distillation under vacuum, the polymer mixture was poured into foil-lined trays and placed into a vacuum oven (−1020 mbar) at 100 °C (HT) or 85 °C (LT) for 3 h to remove the residual solvent and nonpolymerized monomer. The comonomer and initiator formulations for all polymers synthesized throughout this research are detailed in Table 1.

## Results and Discussion

**Figure 2:**
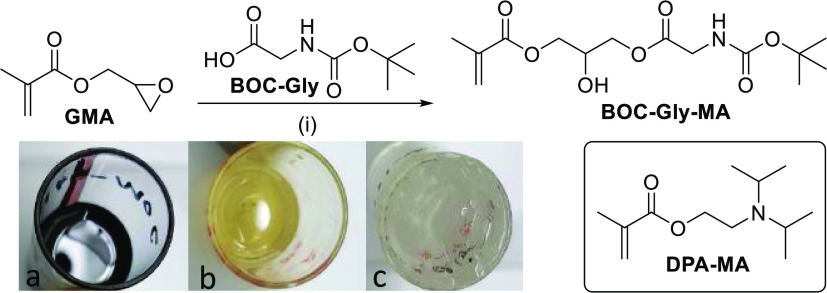
Synthesis of BOC-GLY-MA and the Structure of DPA-MA. (i) K_2_CO_3_, THF. Degree of discoloration from original reaction conditions (a) (dark-colored) to purification using activated carbon filtration (b) (pale-yellow) and finally optimization of reaction conditions to eliminate the color (c) (clear).

To study the concept of incorporating an amine functionality in epoxide-based acrylic powder coatings, two monomers were selected for investigation (Figure 2). 2-(Diisopropylamino)ethyl methacrylate (DPA-MA) was selected as a basic amine functional monomer, whereby the significant steric hindrance offered by *N*-diisopropyl functionality was chosen to suppress nucleophilic addition to epoxide groups, perhaps allowing copolymerization under radical-initiated conditions. To study alongside DPA-MA, we synthesized a *tert*-butylcarbamate (BOC) functional methacrylic monomer from readily available starting materials. We selected the amino acid glycine for this purpose as the BOC-protected amine functional building block.^42^ BOC-protected amino acids are commonly used in polypeptide synthesis in the biopharmaceutical industry, and as such, BOC-amino acids are commercially available.^43−45^ We looked to exploit the widely known carboxylic acid/epoxide ring opening reaction to allow the reaction of *N*-*tert*-butylcarbamate glycine (BOC-Gly) and glycidyl methacrylate (GMA, Figure 2), to synthesize a new monomer (BOC-Gly-MA) containing both the methacrylate functionality (for radical polymerization) and BOC functionality (to study deprotection under filiform corrosion conditions).

BOC-Gly-MA was synthesized using base-catalyzed ring opening of GMA (Figure 2). Conducting this reaction at 80 °C resulted in significant discoloration during the synthesis (Figure 2, image a). As BOC-Gly-MA was intended for copolymerization and applications in high-clarity, transparent powder coatings, discoloration could not be tolerated. Initial attempts to remove the color through activated carbon filtration^46^ was only partially successful (Figure 2, image b), so an investigation was conducted to identify the source of color formation. Chromatographic separation of the reaction components revealed that mixed aromatic derivatives (of *tert*-butyl hydroquinone) were the likely cause of the discoloration, formed at the elevated reaction temperature (80 °C).^47−49^ It was found that by reducing the reaction temperature to 50 °C and increasing the mole percentage of K_2_CO_3_ from 5 to 10%, the reaction could deliver a 75% yield of BOC-Gly-MA on a multigram scale, as a clear and colorless product (Figure 2, image c). Optimal conditions for the synthesis found that separation of any unreacted GMA from the BOC-Gly-MA product was not necessary since GMA was an ingredient in all of the functional polymers synthesized (all at 28% w/w GMA). The resulting BOC-Gly-MA/GMA mixture was thus used directly in the polymerization reactions, and the additional GMA was conveniently added to achieve the target concentration of 28% w/w. The BOC-Gly-MA functional monomer was copolymerized with methyl methacrylate (MMA), glycidyl methacrylate (GMA), *iso*-bornyl methacrylate (*i*BOMA), and styrene (STY) at 125 °C (HT conditions). MMA was selected due to the high-clarity polymers it can produce combined with a relatively low monomer cost. GMA provides the polymer with reactive functional groups capable of forming a thermoset (network) polymer on reaction with an aliphatic diacid (sebacic acid). *i*BOMA is used to increase the *T*_g_ of the final polymer (and resulting) coating so that it would remain in the glassy state at the desired operating temperature. Finally, a low level of styrene (4 wt %) is included to improve the final mechanical properties of the coating.^50^

**Figure 3:**
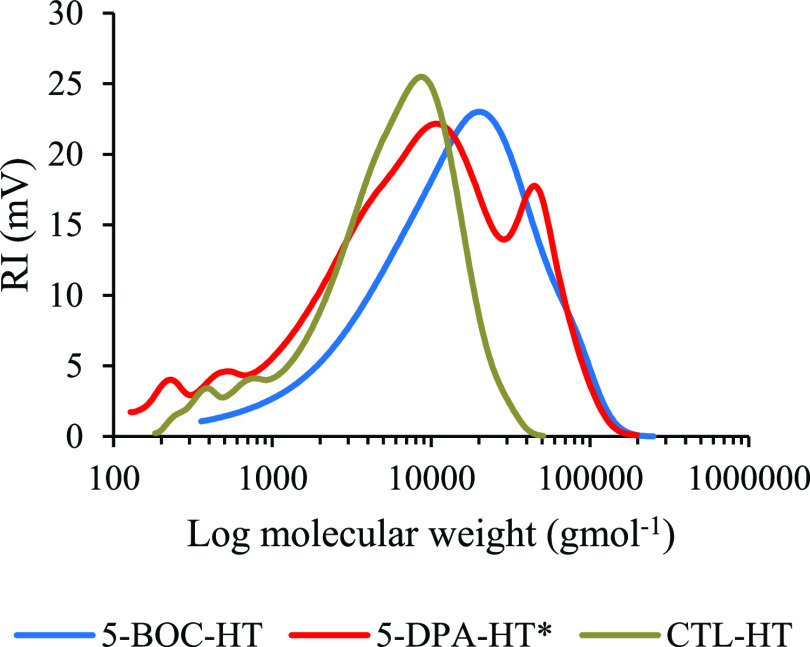
GPC chromatogram overlay of the control, 5-DPA-HT*, and 5-BOC-HT polymers.

**Scheme 1:**
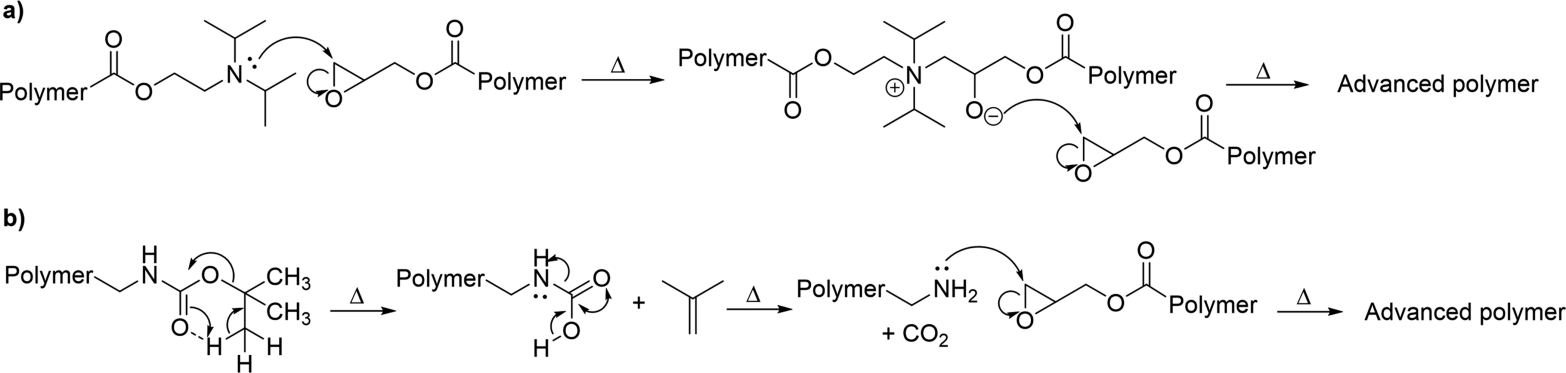
(a) Proposed Mechanism for Advancement of Copolymers of DPA-MA and GMA and (b) Proposed Mechanism for Advancement of Copolymers of BOC-Gly-MA and GMA

The polymerization product (Table 2, entry 3, 5-BOC-HT) was studied by GPC (Figure 3), which showed a significantly increased *M*_n_ and *M*_w_, partnered with a significant increase in polydispersity (PD) illustrated by peak broadening when compared to the control polymer (Table 2, Entry 1, CTL-HT), which did not contain BOC-Gly-MA. This result can be attributed to the presence of the BOC-Gly-MA in the copolymerization with GMA, promoting polymer-to-polymer bond forming reactions (known as advancement), causing the observed increase in *M*_w_ and resulting polydispersity (Table 2, entry 1 vs 3). Polymer-to-polymer bond forming reactions increase both the polydispersity and average functionality (i.e., the average number of reactive functional groups per polymer chain) of the resulting polymer chains. An increase in functionality results in a reduction in the critical extent of the reaction required for gelation (*p*_gel_, as described by Flory and Stockmayer),^51,52^ which in turn impacts flow and coalescence in powder coatings. Poor coalescence in powder coatings negatively impacts both the aesthetic appearance and mechanical properties.^53^ The polymerization of the hindered tertiary amine monomer DPA-MA resulted in a further increase of polymer-to-polymer bond formation, as characterized by a very high polydispersity of 7.95 and emergence of a second, higher molecular weight peak in the GPC chromatogram, characteristic of advancement (Figure 3). The high polydispersity of the copolymer containing 5% w/w DPA-MA monomer (Table 2, entry 8) is attributed to the failure of the diisopropyl functionality to provide sufficient steric hindrance to prevent the reaction with the coexisting epoxide groups (from the GMA monomer) at high polymerization temperatures of 125 °C. The tertiary amine of the diisopropylamine functional groups participates in nucleophilic ring opening with the coexisting epoxide groups on adjacent polymer chains, resulting in polymer-to-polymer bond formation (i.e., advancement, as illustrated in Scheme 1). Tertiary amines are known to promote anionic polymerization via this mechanism, leading to further epoxide ring opening reactions, resulting in the formation of a high-polydispersity polymeric mixture.^54,55^ The *tert*-butylcarbamate groups (BOC) are not typically regarded as nucleophilic, so it was proposed that some of the BOC protecting group must be removed during the high-temperature conditions of polymerization, liberating a primary amine, which would then undergo a facile reaction with the epoxide groups also present in the formulation,^56,57^ resulting in polymer-to-polymer bond formation and increased polydispersity (Scheme 1). To study the proposed thermally initiated BOC removal, a series of model studies were designed to understand the effect of the temperature on BOC deprotection.

**Table 2:** Summary of GPC and DSC Analysis of the Range of Polymers Synthesized in This Study^a^

entry	sample	*M_n_*	*M*_w_	PD	*T*_g_ (°C)
1	CTL-HT	2730	8596	3.15	66.1
2	CTL-LT	3190	7615	2.39	58.8
3	5-BOC-HT	6062	23,383	3.86	67.0
4	5-BOC-LT	3033	7513	2.48	54.0
5	CTL-HT*	2654	7647	2.88	67.7
6	5-BOC-HT*	2591	8717	3.36	57.5
7	5-BOC-LT*	3065	5387	1.76	53.8
8	5-DPA-HT*	2281	18,138	7.95	55.1

a * denotes kilo-scale synthesis. All others are 5 g-scale synthesis.

**Figure 4:**
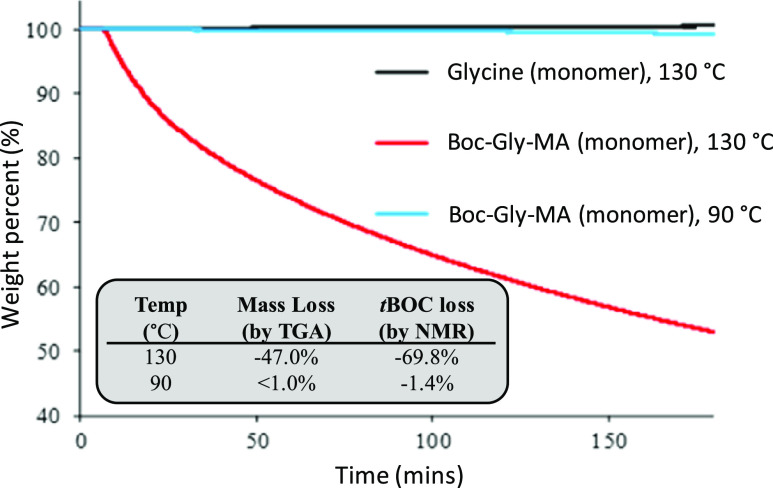
Mass loss study using TGA and (inset) calculated final mass loss (TGA) and estimated *tert*-butylcarbamate (BOC) loss by ^1^H NMR. HT = 130 °C and LT = 90 °C.

As the removal of the BOC group produces CO_2_ and isobutene, (both of which are volatile gases), thermogravimetric analysis (TGA) was used to study the effects of temperature on BOC group retention/removal. A series of experiments were conducted using BOC-protected glycine (BOC-Gly) as a (nonpolymerizable) model for the BOC-Gly-MA. To mimic the conditions of thermally initiated radical polymerization, BOC-Gly was held at either 130 or 90 °C for 3 h in a TGA, and the mass loss was recorded over time. The results of the TGA experiment presented in Figure 4 show a 47% reduction in the mass of BOC-Gly at 130 °C, over the 3 h duration of the experiment. When exposed to lower temperature conditions of 90 °C for 3 h, there was no detectable loss of mass, indicating that BOC functional groups are stable under these conditions. Glycine was also tested as a control where it was exposed to 130 °C for 3 h and did not show any mass loss, confirming that the mass loss was related to the presence of the *tert*-butylcarbamate group.

^1^H NMR was also used to analyze the reaction product after the TGA experiments (Figure 4, inset). Integration of *tert*-butyl ((CH_3_)_3_) against H_2_NCH_2_– allowed an estimation of the degree of BOC protection group removal when heated (see the Supporting Data for details). Comparison of the BOC-Gly-HT (130 °C) reaction product with the glycine control confirmed the presence of glycine (Gly) in the reaction product, formed via thermal deprotection of the BOC group. The BOC-Gly-LT (90 °C) reaction, in contrast, showed no sign of glycine formation, supporting the hypothesis that the BOC functional group remained intact at this lower temperature.

These results suggest that a reduction in polymerization temperature to 90 °C should allow copolymerization of BOC-Gly-MA and GMA, while minimizing BOC deprotection and associated polymer-to-polymer bond formation (as depicted in Scheme 1b). The polymer 5-BOC-LT was therefore synthesized using 5 wt % BOC-Gly-MA and 28 wt % GMA (Table 1, entry 4) at 90 °C. ^1^H NMR analysis (Supporting Information, Table S9) showed that the polymer 5-BOC-LT suffered minimal (<8%) loss of BOC groups, which compared favorably to 5-BOC-HT (Table 1, entry 3), which suffered 28% loss of BOC. This loss of BOC functionality directly affects the polydispersity of the functional polymer as shown in Table 2. The polymer 5-BOC-HT (Table 2, entry 3) showed a polydispersity of 3.86, which decreased to 2.48 (Table 2, entry 4, 5-BOC-LT) at the lower temperature of polymerization. The 5-BOC-LT (Table 2, entry 4) also compares favorably with the control polymers containing 28 wt % GMA only (0% BOC-Gly-MA), which showed a very similar polydispersity of 2.39 under low-temperature (LT, 90 °C) conditions (Table 2, entry 2, CTL-LT). This result shows that at 90 °C, there is no significant impact on polydispersity of using BOC-Gly-MA in a copolymerization with GMA.

A reduction in *T*_g_ could also be seen when comparing LT to HT control polymers by DSC (−7.3 °C), which is also mirrored in the functional polymers, with a loss of 13 and 3.7 °C in *T*_g_ of small- and kilo-scale polymers, respectively. This reduction in *T*_g_ can be explained by the reduction of polymer advancement (shown by reduction in PD), which can be attributed to a reduced number of chain-to-chain bond forming reactions, increasing the rotational freedom and resulting free volume of the polymer chains, thus decreasing the *T*_g_.^58^ As functional monomer concentration in the polymer formulation is increased, the concentration of MMA is decreased proportionately, which can also contribute to a reduction in *T*_g_ by DSC (poly(methyl methacrylate) has a relatively high *T*_g_∞). This can be seen when comparing the *T*_g_ (by DSC) of the CTL-LT (Table 2, entry 2) and the nonadvanced functional polymers synthesized under LT conditions (Table 2, entries 4 and 7).^59^

As powder coating manufacturing requires significant quantities of polymer for formulation, melt extrusion, milling, sieving, and spraying, 5-BOC-HT, 5-BOC-LT, 5-DPA-HT, and CTL-LT were manufactured on a 1 kg (final weight) scale (denoted by * in entries 5–8, Table 2). Using slow monomer addition (starved feed) to control the internal reaction temperature, the 5-BOC-LT* polymer was synthesized on a 1 kg scale, delivering the lowest polydispersity product so far (PD = 1.76, Table 2, entry 7), which compared very favorably to the 5-BOC-HT* polymer with a high polydispersity of 3.36 (Table 2, entry 6). The 1 kg synthesis of 5-DPA-HT* was manufactured for comparison, resulting in the highest polydispersity product (PD = 7.95, Table 2, entry 8), as a result of epoxy ring opening promoted by diisopropyl functional groups (as depicted in Scheme 1a). To study the effect of BOC-Gly-MA and DPA-MA functional polymers on filiform corrosion performance, the CTL-HT*, 5-BOC-HT*, 5-BOC-LT*, and 5-DPA-HT* (Table 2, entries 5–8) polymers were formulated into powder coatings, which were subsequently sprayed onto cast aluminum panels (A356 alloy, in line with aluminum alloy wheels), mechanically scribed, and subjected to accelerated filiform corrosion testing according to the procedure SAE J2635 (see the Supporting Data for details). Analysis of corrosion was conducted using a Quantiz analytical system to measure the filiform corrosion (FFC) track length, FFC track number, FFC width, and total corroded surface area (CSA).

**Figure 5:**
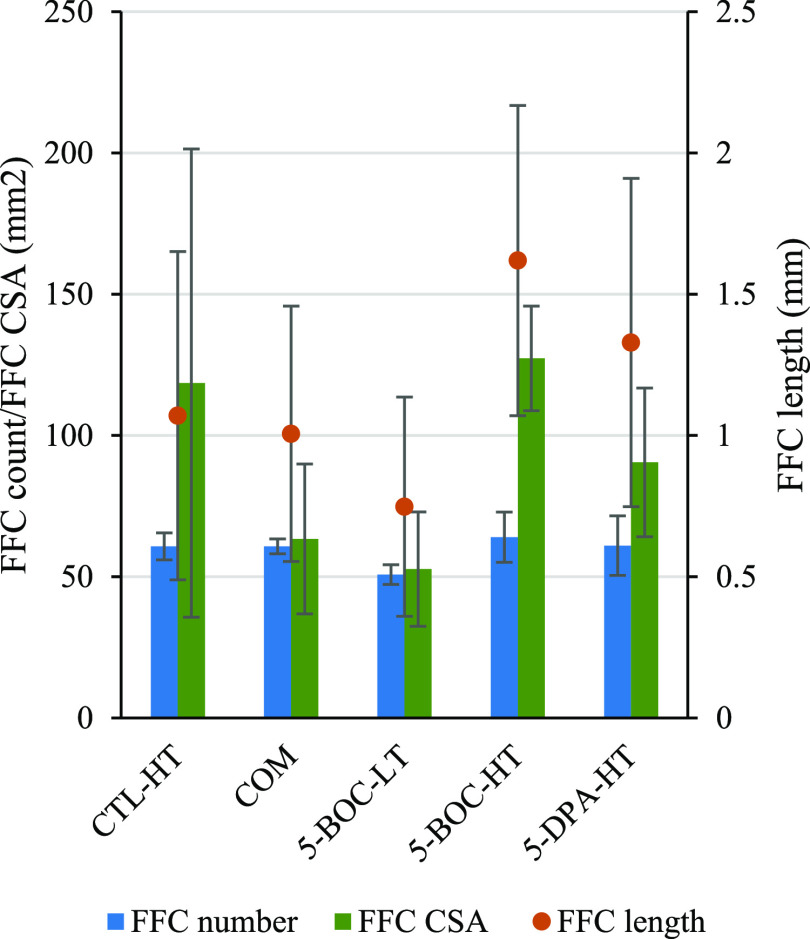
Results of FFC length, FFC count (number), and average CSA from powder-coated aluminum subjected to corrosion testing. Standard deviation was used to calculate the error for FFC number and CSA as the data were normally distributed, while MAD was used to calculate the error for the non-normally distributed FFC length.

The results in Figure 5 (FFC length, orange sphere) show that a powder coating manufactured from 5-BOC-LT* had the lowest average FFC track length (0.748 mm, Figure 5, entry 3), with a combination of Kruskal–Wallis and Dunn’s posthoc testing, proposing that a coating which contained 5% w/w BOC-MA functional monomer and synthesized using LT polymerization conditions (5-BOC-LT*) is more likely to display a significantly shorter average FFC track length than that of coatings synthesized using HT polymerization conditions or containing an alternate functional monomer (i.e., 5-BOC-HT*, 5-DPA-HT*) (see the Supporting Data for details). When comparing the results of average FFC number (count) per panel (FFC count, blue bar, Figure 5), coatings based on 5-BOC-LT* were shown again to improve anticorrosion performance compared to 5-BOC-HT*, control-HT (CTL-HT), and even a standard commercial acrylic coating (COM, see Figure 5, entry 3 vs entries 4, 1, and 2, respectively). Finally, when comparing the coatings’ average corroded surface area (CSA, green bar, Figure 5), it can be seen that there is a substantial difference in corrosion performance from the powder coating based on 5-BOC-LT* (Figure 5, entry 3, CSA = 52.7 mm^2^) in comparison with those based on 5-BOC-HT* (Figure 5, entry 4, CSA = 127.3 mm^2^) and 5-DPA-HT* (Figure 5, entry 5, CSA = 90.5 mm^2^).

The corrosion results show that in the powder coatings where the parent polymers have identical monomer composition (and accompanying “reactive” functionality, Table 1, entries 7 and 8, 5-BOC-LT* vs 5-BOC-HT*), advancement, induced by *in situ* deprotection of *tert*-butylcarbamate groups (BOC), has a substantial and detrimental impact on corrosion performance, across the three key metrics used in this study (i.e., average FFC track length, total FFC number (count), and average corroded surface area, Figure 5, entries 3 vs 4). The results provide evidence of the importance of optimizing the polymerization reaction conditions to minimize polydispersity induced by advancement. This is particularly important when using multiple “reactive” or functional monomers, particularly when potential polymer-to-polymer bond forming reactions are possible. While it was clear that coatings based on advanced polymers performed less well in corrosion testing than equivalent less-advanced coatings, the root cause of this difference required further investigation. A study of coating material properties was conducted to further understand this effect.

**Figure 6:**
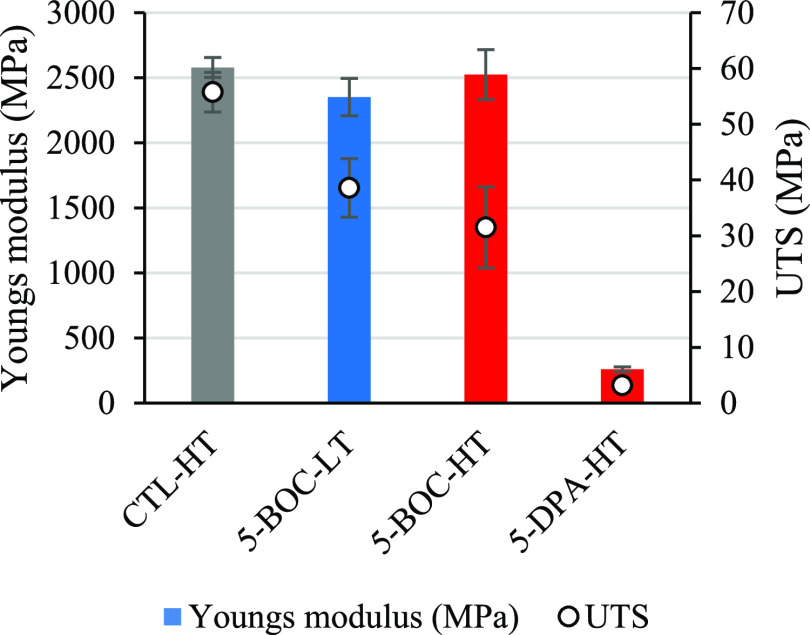
Young’s modulus (YM) and ultimate tensile strength (UTS) results for the four powder coatings based on the large-scale (1 kg) polymerization products.

Powder coatings were prepared as nonadhered “free” films based on the polymers CTL-HT*, 5-BOC-HT*, 5-BOC-LT*, and 5-DPA-HT* (see the Experimental Section 1.2 for details). All coating systems were studied using tensile testing, and the results are illustrated in Figure 6, highlighting Young’s modulus (YM, MPa) and ultimate tensile strength (UTS, MPa). The results showed that coatings based on the polymers 5-BOC-HT* and 5-BOC-LT* measured Young’s moduli of 2524 and 2351 MPa, respectively, which were both similar to the CTL-HT* control coating which lacked the presence of the BOC-Gly-MA monomer (YM = 2578 MPa).

**Figure 7:**
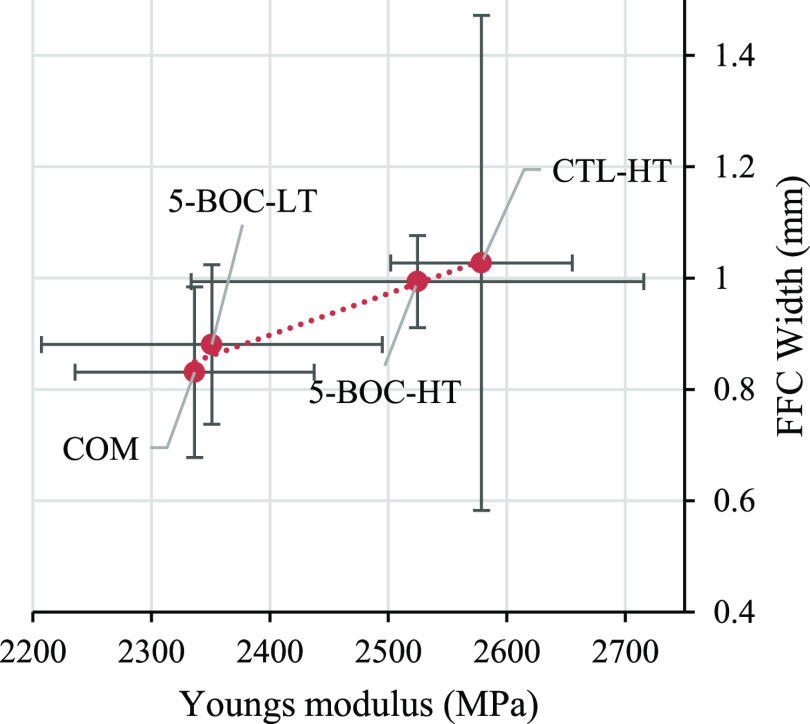
Correlation observed when comparing a coating’s Young’s modulus to the average FFC width.

**Figure 8:**
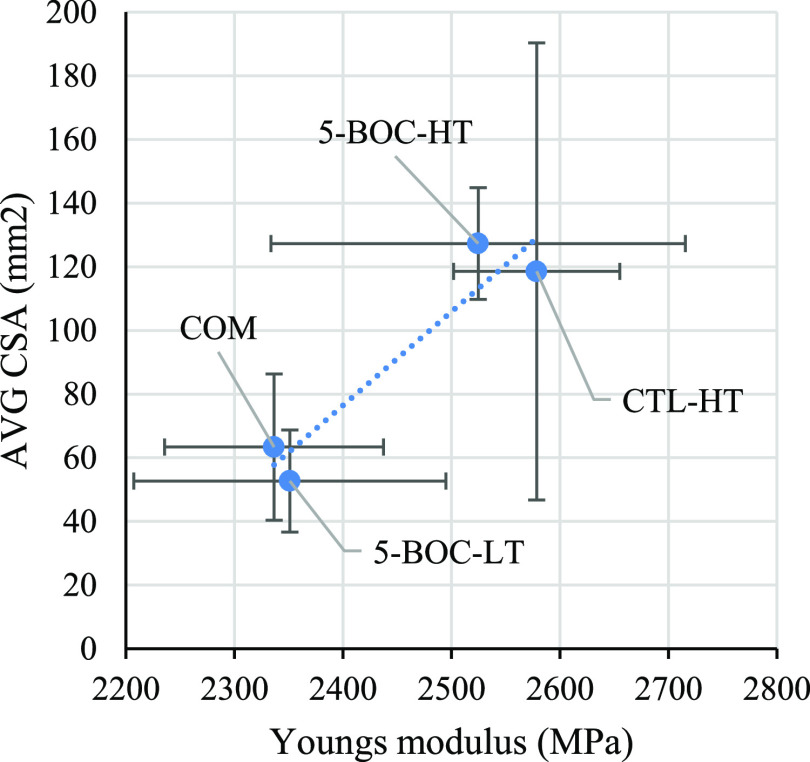
Correlation observed when comparing a coating’s Young’s modulus to the total corroded surface area (CSA).

Coatings based on the most advanced polymer 5-DPA-HT displayed significantly lower ultimate tensile strength values (UTS = 3.16 MPa), when compared to the CTL-HT* control coating (UTS = 55.8 MPa) or the coatings containing the BOC-Gly-MA monomer (5-BOC-LT* UTS = 38.6 and 5-BOC-HT* UTS = 31.5). Generally, coatings based on the highly advanced polymer 5-DPA-HT* showed very poor mechanical performance via both metrics, with Young’s modulus of 259.5 MPa and a UTS of 3.16 MPa. This can be attributed to advancement during the polymerization stage, which results in embrittlement of the resulting coating, contributing to poor corrosion performance. The coating metrics found to have the greatest correlation coefficient and *R*^2^ were Young’s modulus and average FFC width (0.9848, 0.9699, Figure 7) and Young’s modulus (MPa) and total corroded surface area (mm^2^) (0.9488, 0.9003, Figure 8). The results displayed in Figures 7 and 8 support the hypothesis that high-Young’s modulus coatings may be more susceptible to filiform corrosion. Previous research noted that the movement of filiform corrosion progresses in a stepwise segmental manner;^60^ as such, it was theorized that as filiform corrosion moved across the aluminum–coating interface, stiffer (high Young’s modulus) coatings may experience a greater segmental area of coating disbondment, linked to FFC width and CSA, while more flexible coatings (low Young’s modulus) were theorized to yield with increased ease, resulting in reduced segments of coating removal, with each step of FFC propagation.

**Figure 9:**
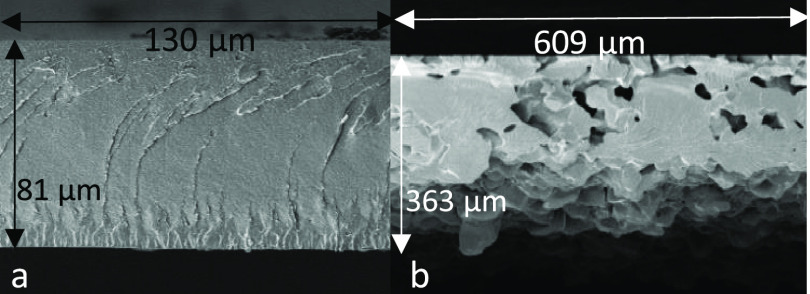
SEM image showing the cross-section of coatings based on (a) 5-BOC-HT* and (b) 5-DPA-HT*. The difference in coating thickness (5-BOC-HT 0.081 mm, 5-DPA-HT 0.363) required an increased field of view for 5-DPA-HT.

Cross-sectional analysis of coatings based on the polymers 5-BOC-HT* (polydispersity = 3.36) and 5-DPA-HT* (polydispersity = 7.95) was conducted by using scanning electron microscopy (SEM) (Figure 9). This study exposed a poor degree of powder particle coalescence in coatings made from the highest-polydispersity (i.e., most advanced) 5-DPA-HT* polymer. During the powder coating application process, heat is applied to the powder-coated substrate, to promote melting, flow, and coalescence of the powder particles into a single cohesive film (i.e., the final coating). Failure of this process (due to the poor particle flow and coalescence) can result in a coating with poor cohesive strength, made from ‘fused/sintered powder particles’ rather than a fully coalesced coating. In the case of coatings based on the 5-DPA-HT* polymer, the very high degree of advancement causes both (i) increased melt viscosity of the 5-DPA-HT* polymer in the molten state and (ii) an increase in weight-average functionality (*f*_w_) of the polymer. High melt viscosity results in a poor flow and coalescence in the melt state, contributing to the poor mechanical performance of coatings based on the 5-DPA-HT* polymer. Increased *f*_w_ results in a decrease in the critical extent of the reaction required for gelation (*p*_gel_);^52^ thus, gelation occurs at a lower extent of reaction, which also limits the opportunity for polymer flow and particle coalescence. Both factors contribute to the porous coating structure for the 5-DPA-HT* polymer shown in Figure 9b, and poor mechanical properties are represented in (Figure 6, entry 4).

We have demonstrated the ability to introduce a *tert-*butylcarbamate (BOC)-protected amine functionality into a multifunctional polymer with GMA while causing minimal advancement (Table 2, entry 7, 5-BOC-LT*). We have also shown that the resulting polymer could be used to form thermoset powder coatings with favorable corrosion performance compared to the existing commercial powder coatings (Figure 5). The next stage of the research was to determine whether acid-mediated *tert-*butylcarbamate (BOC) deprotection of the functional coating occurred under the low-pH environment of a FFC corrosion head, thus supporting pH buffering as the likely mode of action for enhanced corrosion protection. To enhance the concentration of BOC functional groups for analysis, while maintaining the ability to form a thermoset coating, a multifunctional polymer based on 50 wt % BOC-Gly-MA and 28 wt % GMA monomer was synthesized (Table 1, entry 5, 50-BOC-HT) and studied in both solution model tests and coating model tests.

**Figure 10:**
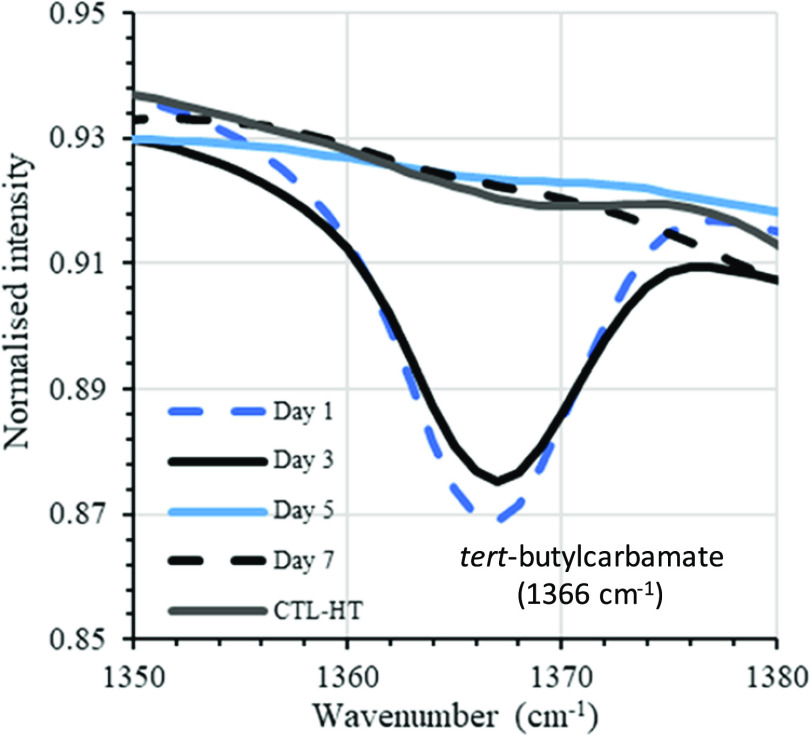
Overlay of IR spectra recorded in transmittance mode between 1350 and 1380 cm^–1^, taken over the 7-day model corrosion test period. Focusing on the area displays the depleting signal produced by the *tert*-butylcarbamate (BOC) group over time.

For solution model testing, the polymer 50-BOC-HT was exposed to conditions designed to mimic the acidic aqueous environment of the filiform corrosion process. Accordingly, 4 M HCl (aq.)/THF (1:1 v/v) was prepared, and samples of the 50-BOC-HT polymer were submerged into the solution. At each time point (1–7 days), a sample was removed and dried, and changes in the polymer functional groups were studied by infrared spectroscopy. The relative peak intensity of the *tert*-butylcarbamate (BOC) signal at 1366 cm^–1^ was compared to the (hydrolytically stable) aliphatic polymer backbone −CH_3_ and −CH_2_– bond signal at 1470–1430 cm^–1^, to measure the reduction in BOC protecting group concentration over the 7-day period (see the Supporting Data for details). The results showed that the concentration of BOC protecting groups was significantly reduced over the 7-day study period, observed through reduction in peak intensity at 1366 cm^–1^, when compared to a control experiment under neutral (nonacidic, pH = 7.0) conditions (Figure 10). This result was support by ^1^H NMR analysis over the 7-day study, which indicated a 67% reduction in *tert-*butylcarbamate signals (1.44 ppm), relative to polymer backbone −CH_3_ groups (3.73 ppm).

**Figure 11:**
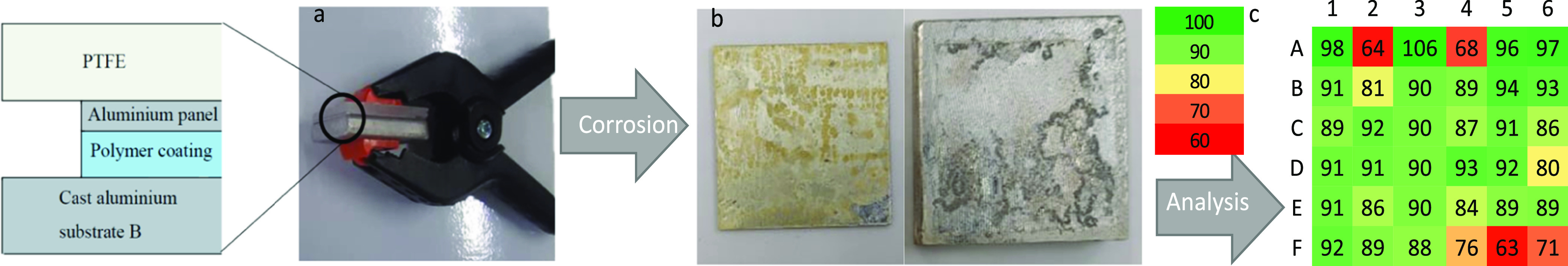
(a) Assembled clamped sample of the 50-BOC-HT coating prior to subjection of accelerated corrosion conditions; (b) disassembled clamped sample after CASS exposure and 30 days in a humidity chamber; and (c) grid with values representing the percentage of BOC groups remaining.

Studying the concentration of the BOC functionality at the coating–metal interface was more challenging. Accordingly, the 50-BOC-HT polymer was formulated and cured into a thermoset coating and applied on a 25 mm × 25 mm aluminum Q-panel (Figure 11a, “aluminum panel”). The coated Q-panel was placed (coated face down) onto an aluminum A356 alloy panel (Figure 11a, “cast aluminum substrate B”), prepared according to the casting and machining process of an aluminum alloy wheel. The substrates were then clamped together according to the illustration in Figure 11a. The resulting test unit was exposed to a corrosion process of 6 h of copper accelerated-acetic acid salt spray (CASS), before placing in a humidly and temperature-controlled chamber (85% relative humidity at 60 °C) for 28 days to promote filiform corrosion from the exposed edge. The samples were then disassembled (see Figure 11b), and the coating surface (previously in contact with “cast aluminum substrate B”) was analyzed using an infrared spectroscopy microscope. The relative abundance of *tert*-butylcarbamate (1395–1385 cm^–1^) was collected by infrared spectroscopy vs a baseline value obtained (in triplicate) prior to exposure to the corrosion testing and presented as a 6 × 6 grid (Figure 11c, green illustrates no change within the standard deviation of the baseline samples, yellow to red indicates a percentage reduction in *tert*-butylcarbamate concentration on the coating surface).

Analysis indicates that the greatest reduction in *tert*-butylcarbamate concentration (37%) was more often found around the sample edge; in contrast, little or no change in *tert*-butylcarbamate concentration was observed in the sample center. The sample edge in this experiment is analogous to an area of coating that has sustained damage, which is the initiation site for the FFC displayed in Figure 11b. The aqueous acidic environment caused by the propagating FFC trails was proposed to be the cause for the *tert*-butylcarbamate concentration reduction. These results align with the hypothesis that the aqueous low-pH environment created in the presence of filiform corrosion is sufficiently acidic to remove *tert*-butylcarbamate groups at the aluminum–coating interface, liberating the amine functionality, which will increase the local pH due to their basic nature. These results also align with those of the solution model study.

## Conclusions

It has been shown that amine-containing monomers, containing a *tert-*butylcarbamate functionality, are able to be copolymerized with the epoxy-functional monomer GMA and formulated into a thermoset powder coating. The introduction of the BOC-Gly-MA monomer was shown to improve a coating’s anticorrosion performance (5-BOC-LT*), presenting the lowest average FFC track length, total FFC number (count), and total CSA, vs other experimental coatings in this study. Solution and coating models were designed to study the effect of filiform corrosion on the *tert-*butylcarbamate functional polymer and specifically at the coating–aluminum interface. The study provided evidence to support the hypothesis that the *tert-*butylcarbamate protecting group can be removed from the polymer coating surface under the low-pH (acidic) conditions of filiform corrosion, with the liberation of a basic primary amine, proposed to be the source for the material’s improved anticorrosion performance.

It was shown that when polymerization conditions are not optimized (HT), polymer advancement can occur through the formation of polymer-to-polymer bond forming reactions, inhibiting the polymer’s ability to form a cohesive coating. The presence of advancement was then shown to be detrimental to a coating’s material strength, which resulted in a decrease in corrosion performance, with analysis finding that an increase in a coating’s Young’s modulus (increase in coating stiffness) resulted in an increase in the total CSA linked to increasing FFC width.

## Supporting Information Available

Spectral data and extended methodologies for synthesis; application; and testing processes (PDF)
