# Supplementary material for: pH-Responsive, Thermoset Polymer Coatings for Active Protection against Aluminum Corrosion
Source: ACS Appl Mater Interfaces. 2024 Mar 1;16(10):12986–95. doi: 10.1021/acsami.3c14752 (PMC10941078; doi:10.1021/acsami.3c14752)
Supplement: Supplementary file 1 — am3c14752_si_001.pdf [file am3c14752_si_001.pdf]

Supporting information

# pH-responsive, thermoset polymer coatings for active protection against aluminum corrosion

Joseph Watson[a], Victoria Balmforth[b], Elaine Gray[b] and Matthew G. Unthank*[a]

AUTHOR ADDRESS

a: Northumbria University, Newcastle upon Tyne NE1 8ST, UK. matthew.unthank@northumbria.ac.uk

b: AkzoNobel, Polymer Development Group, Stoneygate Lane, Felling, Tyne & Wear, NE10 0JY, UK.

*: Department of Applied Science, Northumbria University, Newcastle upon Tyne NE1 8ST, UK. matthew.unthank@northumbria.ac.uk

* Corresponding author.

## 1.1 Materials

All materials were purchased from commercial vendors listed in Table S1 and used as received without further purification for synthesis unless specifically stated.

*Table S1: Reagents used throughout synthesis.*

| Chemical | CAS | Supplier | Purity/Grade |
|---|---|---|---|
| **Monomer synthesis** | | | |
| BOC-Gly | 4530-20-5 | Aldrich | 99% |
| Potassium carbonate | 584-08-7 | Fischer | 99% |
| Glycidyl methacrylate | 106-91-2 | Acros organics | 97% |
| **Polymer synthesis** | | | |

| | | | |
|---|---|---|---|
| Methyl methacrylate | 80-62-6 | Acros organics | 99% |
| Styrene | 100-42-5 | Acros organics | 99% |
| Isobutyl Methacrylate | 97-86-9 | Fisher | 85-90% |
| 2-(Diisopropylamino)ethyl methacrylate | 16715-83-6 | Aldrich | 97% |
| Trigonox 21 S | N/A | AkzoNobel | 99% |
| 2,2′-Azobis(2-methylpropionitrile) | 78-67-1 | Aldrich | 98% |
| Butyl acetate | 123-86-4 | Fischer | 99.5% |
| Isopropyl acetate | 108-21-4 | Fischer | For synthesis |
| **Solvents/Reagents** | | | |
| Tetrahydrofuran | 109-99-9 | Fischer | 99% |
| Ethyl acetate | 141-78-6 | Fischer | 99.5% |

All polymer coatings were either supplied by AkzoNobel, or were manufactured into powder coatings at AkzoNobel.

## 1.2 Powder coating manufacture

The acrylic polymers were manufactured into powder coatings, according to the standard powder formulation shown below.

*Table S2: Formulation ingredients for a powder coating*

| Purpose | Reagent | WT% |
|---|---|---|
| Binder/Resin | Acrylic resin | 81.1 |
| Crosslinking agent | Sebacic acid | 16.7 |
| Particle flow aid | Aeroxide Alu C | 0.2 |
| Degasser | Benzoin | 1.0 |
| Polymer melt flow aid | Resiflow PL-200 | 1.0 |

The formulation was then melt mixed using a twin-screw extruder with 8 heating zones. The first 4 zones were heated to 20 °C to blend and mix the coating, the final 4 zones were heated to 100 °C to homogenize the coating. A short residence time (30-60 seconds) is used to prevent premature curing during the mixing process, where the screw speed is increased from 200RPM at polymer feed to 400RPM after feed is complete. Once the homogeneous polymer/additive blend has exited the extruder, it is cooled and once again kibbled into a coarse grain before grinding takes place, it is at this stage further additives are incorporated into the powder coating blend. The grain is milled to a fine powder with a target maximum particle size of 125 μm. The fully formulated powder coating, is then sprayed onto earthed aluminum test panels using a corona spray application process. Each panel was coated with 1.3±0.1 g of powder, the coated aluminum samples were then cured by first heating to 205 °C for 5 minutes then 185 °C for a further 25 minutes.

Thin (61-363 μm) free-films of cured thermoset coatings, unadhered to any metal were produced by first treating aluminum Q-panels (100 mm X 150 mm) with a release agent (Marbocote 227 CE (based on C7-C9, iso-alkanes)) following manufacturer's specifications. Once the release agent was applied to an aluminum substrate, powder coatings could then be sprayed onto the surface and cured following the same methods detailed above. Free-film samples were then cut to the specifications required using custom settings in the Glowforge laser cutter software of 60% power and a speed of 370.

## 1.3 Analytical methods and equipment

### 1.3.1 Mass Spectroscopy (MS)

Samples were acquired using a Thermo RSLC coupled to an ABSciex 6600 QTof. The equivalent of 1-5 µM (1-5 pmol/µl) was injected onto the system per run. The loading pump was used at high flow to run linear chromatography gradients. All samples were acquired in positive mode. The buffer system comprised of buffer A 0.1% (v/v) formic acid and buffer B 99.9% acetonitrile, 0.1% (v/v) formic acid. Chromatographic separations were achieved using a Fortis C8 column, 100 mm x 2.1 mm, 3 µM particle size, 45 °C at a flow rate of 250 µl/min. Samples were loaded onto the column and desalted online for 1 minute, with eluent diverted to waste. A valve switch directed flow to the mass spectrometer. Samples were then eluted from the column using a linear gradient from 3- 70% Buffer B over 12 min. Total run time was 17 min. The eluent was directed into an ABSciex 6600 QTof, operated in positive mode. Source conditions were; temperature 180 °C, GS1 25, GS2 15, ISFV 4500v. Data was acquired in MS scan mode between 100-1000 m/z. Resolution of the instrument was 45,000 at m/z 829.54

**High-resolution mass spectra**

Results recorded using a Vanquish liquid chromatography front end connected to IDX high resolution mass spectrometer system. The chromatographic separation was achieved using a Waters Acquity UPLC BEH amide column (2.1 x 150 mm with particle size of 1.7 µm) part no 186004802, operating at 45 °C with a flow rate of 200 µL/min. MS data were acquired using the AcquieX acquisition workflow.

### 1.3.2 Differential Scanning Calorimetry (DSC)

Glass transition (Tg) analyses were conducted on all polymeric materials using a Perkin-Elmer Pyris DSC 8500 and analyzed on Pyris software (version 11.1.1.0492). Samples had previously been thermally cured in an oven at 170 °C. Sample masses of 3-6 mg were weighed into standard aluminum DSC pans with perforated lids and were heated from -50 °C to 200 °C at a constant rate of 20 °C per minute (unless otherwise stated) and the Tg reported as the midpoint of the endothermic step in the heat flow signal

output (Tg onset and endpoint was also recorded). Melting point analysis was conducted using a PerkinElmer Pyris DSC 8500 with an intercooler II and analyzed on Pyris software (version 11.1.1.0492). Solid samples between 3-6 mg were weighed into standard aluminum DSC pans and heated from -50 °C to 300 °C at a constant rate of 20 °C per minute and the melting point taken from the peak of the endothermic event that corresponds to the melting transition on the heat flow signal output.

### 1.3.3 SEM imaging

Was recorded using a field emission TESCAN MIRA 3 with gigantic chamber at 5kV, analyzed using Alicona 3d imaging and AztecEnergy. The samples analyzed were prepared by first cooling a 10 x 100mm section of coating free-film in liquid nitrogen, the cooled sample was then fractured. The edge of the samples fracture point was coated in silver to increase sample conductivity.

### 1.3.4 Microscopic infra-red spectra

Were recorded from 4000 to 650 $cm^{-1}$ in 16 scans using a PerkinElmer Spotlight 150i FT-IR microscope, in ATR mode with an aperture of 100 x 100µm.

### 1.3.5 Statistical analysis

Was performed using JASP software, further details of the results produced are later discussed.

## 1.4 Monomer experimental

BOC-Gly-MA

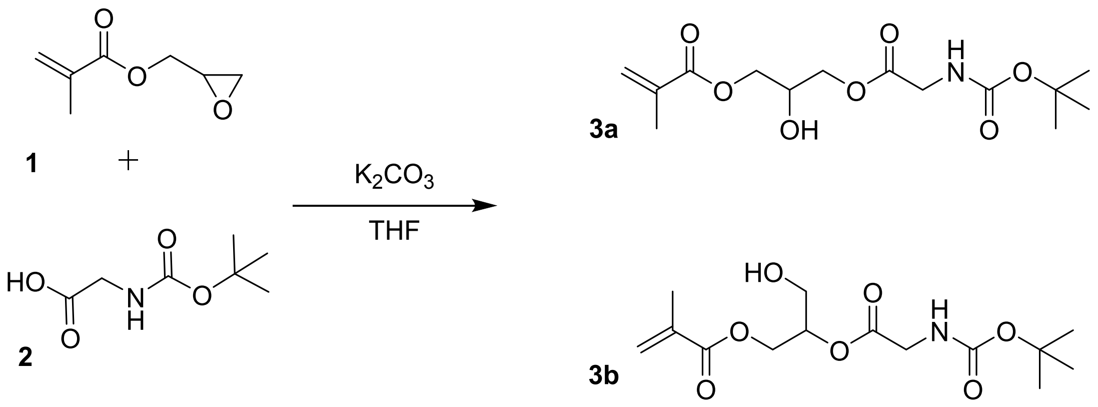


To a solution of BOC-GLY (**2**) (14.73 g, 84 mmol) and $K_2CO_3$ (0.97 g, 7 mmol) in THF (20 mL, degassed using $N_2$) was added GMA (**1**) (9.95 g, 70 mmol) before heating to 50 °C under stirring and $N_2$. After 24 hours the reaction mixture was cooled to room temperature and diluted with ethyl acetate (100 mL). The reaction mixture was washed with a saturated $NaHCO_3$ solution (3 x 100 mL), the organic phase was collected, and dried over $MgSO_4$. The organic phase was then concentrated under reduced pressure, affording the resulting clear oil **3** as a mixture of isomers (**$3_a$** & **$3_b$**) (19.98 g 75%), as determined by $^1$H-NMR and mass spectrometry.

**$3_a$**: $\delta_H$ (400 MHz; $CDCl_3$): 6.13 (s, 1H), 5.61 (s, 1H), 5.03 (s, 1H), 4.31-4.19 (m, 4H), 4.17-4.12 (m, 1H), 3.93 (d, J = 5.5 Hz, 2H), 2.73 (s, 1H), 1.94 (s, 3H), 1.44 (s, 9H)

**$3_a$**:$\delta_C$ (400 MHz; $CDCl_3$): 170.5 (C=O), 167.4 (C=O), 156.2 (C=O), 135.8 (C=C), 126.5 (C=C), 80.3 (**C**($CH_3$)), 67.8 (C-O), 66.0 (C-O), 65.1 (C-O), 42.4 ((C=O)**C**NHR), 28.3 (C(**C**$H_3$)), 18.3 (**C**$H_3$)

$\nu_{max}$/cm$^{-1}$ = 3380w (broad), 2975w, 1703s, 1520m, 1367w

MS: (+ESI) ([M+H]) major = *m/z* 318.155, minor = *m/z* 318.155

*Table S3: Optimization of BOC-Gly-MA reaction conditions. Where sample A, B, and C refers to the images displayed in Figure 2 (main text).*

| Sample | $K_2CO_3$ (mole %) | Temperature (°C) | Colour |
|---|---|---|---|
| **A** | 5 | 80 | Dark Red |
| **B*** | 5 | 80 | Pale yellow |
| **C** | 20 | 50 | Colorless |

*Sample was also treated with activated charcoal and filtered.

## 1.5 Powder coating curing conditions

Powders were sprayed to a total adhered powder mass of 1.3 ± 0.1 g onto a 70 mm by 150 mm aluminum Q-panel for corrosion testing, or 100 mm X 150 mm for free film creation. The coated panels were then heated in a conventional fan-oven to 205 °C for 5 minutes, followed by 185 °C for a further 25 minutes.

## 1.6 Polymer synthesis

### 1.6.1 General method of small-scale polymer synthesis

**High temperature (HT) and low temperature (LT) small scale polymerisation reaction conditions**

A solution of mixed acrylic and methacrylic monomers (according to Table 1, main text) was combined with either *tert*-butyl peroxy-2-ethylhexanoate (Trigonox 21S) (high-temperature, HT method) or azobisisobutyronitrile (AIBN) (low-temperature, LT method) in butyl acetate (HT) or isopropyl acetate (LT) (solvent mass equal to 20% of the total monomer mass). The resulting solution of monomer and initiator

was added drop-wise to a 100 mL 3-neck round bottom flask containing butyl acetate (HT) or isopropyl acetate (LT) (equal to 50% of the monomer mass) at an internal flask temperature of either 125 °C (HT method) or 85 °C (LT method) by use of a syringe pump at a rate of 0.5 mL/min, whilst under $N_2$ and constant stirring provided by a magnetic stirrer. Once solution addition was complete, the mixture was stirred for a further 1 hour at 125-130 °C (HT) or 85-90 °C (LT), after which time the hot polymer mixture was poured into foil lined trays, and then placed into a vacuum oven (-1020 mbar) at 100 °C (HT) or 85 °C (LT) for 3 hours to remove residual solvent and non-polymerized (residual) monomer.

### 1.6.2 General method of large-scale polymer synthesis

**High temperature (HT) and low temperature (LT) large scale polymerisation reaction conditions**

A solution of mixed acrylic and methacrylic monomers (according to Table 1, main text) was combined with *tert*-butyl peroxy-2-ethylhexanoate (Trigonox 21S) (high-temperature, HT method or azobisisobutyronitrile (AIBN) (low-temperature, LT method) and diluted with butyl acetate (HT and LT) (solvent mass equal to 20% of the total monomer mass). This mixture was added dropwise to a 2 L preheated flange neck flask containing butyl acetate (HT and LT) (equal to 50% of the monomer mass) heated to 125 °C (HT) or 85 °C (LT), by use of a peristaltic pump at a rate of 7.4 mL/min over 3 hours, whilst under $N_2$ and constant stirring provided by an overhead stirrer. Once the solution addition was complete, the reaction was then stirred for a further 1 hour at 125-130 °C (HT) or 85-90 °C (LT). A solution of Trigonox 21S (HT) or AIBN (LT) equal to 10% mass of initial initiator weight was then added to the reaction, where stirring at a temperature of 125-130 °C (HT) or 85-90 °C (LT) was maintained for a further 45 minutes, for the purpose of reacting residual monomer. The reaction was then maintained at 100 °C and a vacuum distillation was performed to remove the majority of the solvent. After 40 minutes of distillation under

vacuum the hot polymer mixture was poured into foil lined trays, the tray containing the polymer was then placed into a vacuum oven (-1020 mbar) at 100 °C (HT) or 85 °C (LT) for 3 hours to remove residual solvent and non-polymerized (residual) monomer.

When formulating polymerisation reaction compositions, the initiator used, and its mole percentage was selected so that Mn and Mw were matched (within experimental error) under the differing processing conditions by i) varying the initiator:monomer ratio and ii) using a lower decomposition temperature initiator for the LT conditions.

The structures of BOC-Gly-MA, DPA-MA, and control (CTL) polymers are shown in Figure S1 to Figure S5. Table S4 to Table S8 provide the weight percent of the monomers, and mole percent of the radical initiator present in each polymer formulation. Where a (T) or an (A) has been used to denoted whether Trigonox 21s or AIBN was used as the radical initiator respectively.

Due to the complex structure of the statistical (random) co-polymers synthesized during this research, steps were taken to simplify the $^{1}$H-NMR analysis. While the polymer chains present in each sample will vary in molecular weight, the molar percentage of the co-monomers present will be representative of the whole polymer sample. As such, each $^{1}$H-NMR has been integrated assuming 100 moles of the sample were present, so the consistent peaks such as that of the -$OCH_3$ of MMA could be used as a reference for further integrations. As it was known that the -$OCH_3$ would produce an integration of 3 per repeating MMA unit, and in a representative formulation MMA makes up 62.8 mol% of the co-polymer formulation, the relative integration for the peak representing -$OCH_3$ would therefore be 188.4 (62.8 x 3).

### 1.6.3 Small scale control polymerization

*Table S4: Monomer and initiator weight and mole percent values used to formulate the small-scale control IP polymers.*

| | Weight percent | | | | Mol % | |
|---|---|---|---|---|---|---|
| Name | MMA | GMA | STY | IBOMA | Radical int | Total mass (g) |
| CTL-HT | 52 | 28 | 4 | 16 | (T) 4 | 5.0 |
| CTL-LT | 52 | 28 | 4 | 16 | (A) 8 | 5.0 |

CTL-HT

$\delta_H$ (400 MHz; $CDCl_3$): 7.21-6.98 (m, 23H), 4.31 (s, 32H), 3.77 (s, 23H), 3.58 (s, 188H), 3.20 (s, 24H), 2.84 (s, 24H), 2.62 (s, 24H), 2.03-0.64 (m, 629H)

GPC analysis: $M_w$ = 7546 $gmol^{-1}$, $M_n$ = 2443 $gmol^{-1}$, PD = 3.09. DSC Tg = 59.5 °C.

CTL-LT

$\delta_H$ (400 MHz; $CDCl_3$): 7.18-7.01 (m, 23H), 4.32 (s, 33H), 3.77 (s, 23H), 3.59 (s, 188H), 3.20 (s, 23H), 2.84 (s, 23H), 2.63 (s, 23H), 2.09-0.641 (m, 633H)

GPC analysis: $M_w$ = 7615 $gmol^{-1}$, $M_n$ = 3190 $gmol^{-1}$, PD = 2.39. DSC Tg = 58.8 °C.

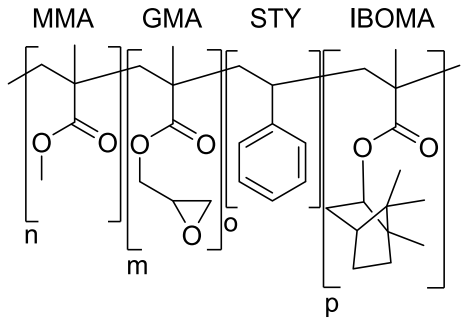


*Figure S1: General structure of the large-scale CTL functional polymers.*

### 1.6.4 Large scale (~1kg) control polymerization

*Table S5: Monomer and initiator weight and mole percent values used to formulate the large-scale control (CTL-HT) polymers.*

| | Weight percent | | | | Mol % | |
|---|---|---|---|---|---|---|
| Polymer | MMA | GMA | STY | IBOMA | Radical int | Total mass (g) |
| CTL-HT* | 0.52 | 0.28 | 0.04 | 0.16 | (T) 0.04 | 1028 |

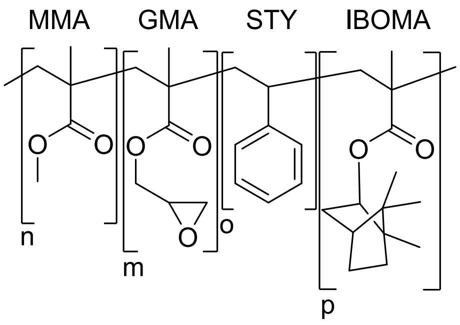


*Figure S2: General structure of the large-scale CTL-HT polymer.*

CTL-HT*

$\delta_H$ (400 MHz; $CDCl_3$): 7.21-7.03 (m, 23H), 4.31 (s, 33H), 3.79 (s, 24H), 3.60 (s, 188H), 3.22 (s, 24H), 2.86 (s, 24H), 2.64 (s, 24H), 2.11-0.86 (m, 630H)

GPC analysis: $M_w$ = 7647 gmol$^{-1}$, $M_n$ = 2654 gmol$^{-1}$, PD = 2.88. DSC Tg = 67.73 °C. TGA decomposition at 208 to 491 °C.

### 1.6.5 Small scale BOC-Gly-MA (3) functional polymers

*Table S6: Monomer and initiator weight and mole percent values used to formulate the small-scale BOC functional polymers.*

| | Weight percent | | | | | Mol % | |
|---|---|---|---|---|---|---|---|
| Name | MMA | GMA | STY | IBOMA | BOC | Radical int | Total mass (g) |
| 50-BOC-HT | 0 | 28 | 0 | 22 | 50 | (T) 4 | 5.0 |
| 5-BOC-HT | 47 | 28 | 4 | 16 | 5 | (T) 4 | 10.0 |
| 5-BOC-LT | 47 | 28 | 4 | 16 | 5 | (A) 8 | 5.0 |

50-BOC-HT

$\delta_H$ (400 MHz; $CDCl_3$): 4.46-3.63 (m, 426H), 3.25 (s, 20H), 2.86 (s, 22H), 2.66 (s, 21H), 2.20-1.57 (m, 421H), 1.46 (s, 235H), 1.25-0.72 (m, 424H). Sample was only sparingly soluble in $CDCl_3$ resulting in poor resolution of peaks.

GPC analysis: $M_w$ = 60,922 gmol$^{-1}$, $M_n$ = 5430 gmol$^{-1}$, PD = 11.16. DSC Tg = 36.2 °C.

5-BOC-HT

$\delta_H$ (400 MHz; $CDCl_3$): 7.20-7.00 (m, 24H), 4.34 (s, 34H), 3.81 (s, 25H), 3.60 (s, 186H), 3.23 (s, 25H), 2.86 (s, 25H), 2.64 (s, 25H), 2.09-0.75 (m, 184H), 1.46 (s, 18H), 1.23-0.75 (m, 452H)

GPC analysis: $M_w$ = 23,383 gmol$^{-1}$, $M_n$ = 6062 gmol$^{-1}$, PD = 3.86. DSC Tg = 67.0 °C.

5-BOC-LT

$\delta_H$ (400 MHz; $CDCl_3$): 7.20-7.01 (m, 24H), 4.32 (s, 34H), 3.80 (s, 24H), 3.60 (s, 186H), 3.22 (s, 25H), 2.86 (s, 25H), 2.64 (s, 25H), 2.00-1.73 (m, 185H), 1.46 (s, 18H), 1.29-0.86 (m, 451H)

GPC analysis: $M_w$ = 7513 $gmol^{-1}$, $M_n$ = 3033 $gmol^{-1}$, PD = 2.48. DSC Tg = 54.0 °C.

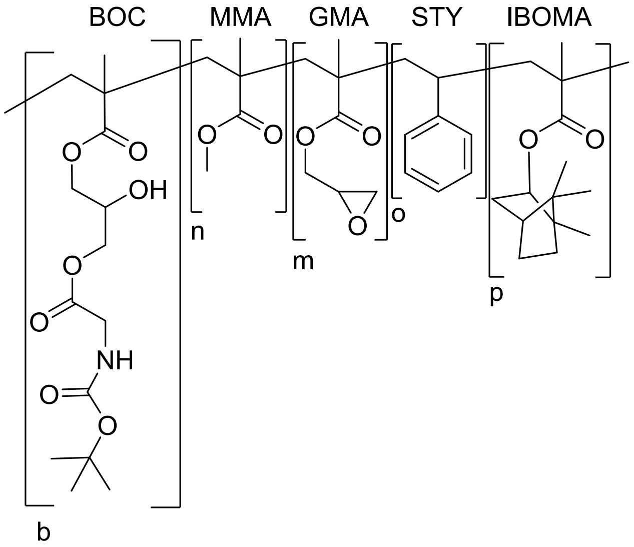


*Figure S3: General structure of the large-scale BOC and GMA functional co-polymers.*

### 1.6.6 Large scale BOC-Gly-MA (3) functional polymers

*Table S7: Monomer and initiator weight and mole percent values used to formulate large scale BOC functional polymers.*

| | Weight percent | | | | | Mol % | |
|---|---|---|---|---|---|---|---|
| Polymer | MMA | GMA | STY | IBOMA | BOC | Radical int | Total mass (g) |
| 5-BOC-HT* | 0.47 | 0.28 | 0.04 | 0.16 | 0.05 | (T) 0.04 | 1031 |
| 5-BOC-LT* | 0.47 | 0.28 | 0.04 | 0.16 | 0.05 | (A) 0.08 | 802 |

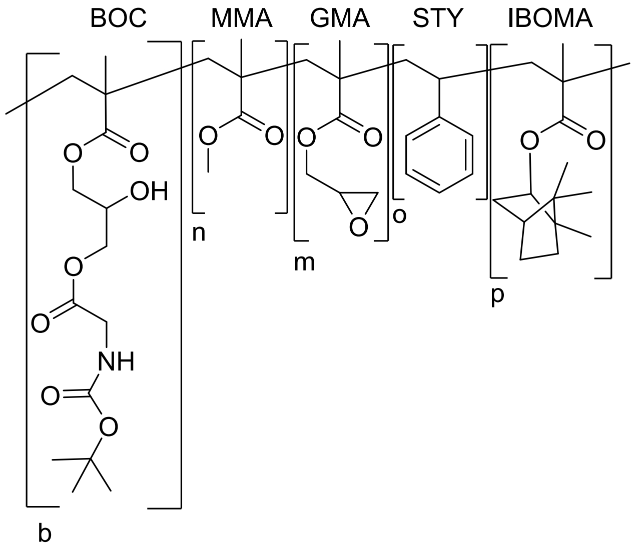


*Figure S4: General structure of the large-scale BOC functional polymers.*

5-BOC-HT*

$\delta_H$ (400 MHz; $CDCl_3$): 7.19-7.00 (m, 24H), 4.33 (s, 34H), 3.81 (s, 25H), 3.58 (s, 190H), 3.22 (s, 22H), 2.84 (s, 23H), 2.62 (s, 22H), 2.17-1.64 (m 128H), 1.45 (s, 13H), 1.31-0.61 (m, 507H)

GPC analysis: $M_w$ = 8717 $gmol^{-1}$, $M_n$ = 2591 $gmol^{-1}$, PD = 3.36. DSC Tg = 57.52 °C. TGA decomposition at 214 to 498 °C.

5-BOC-LT*

$\delta_H$ (400 MHz; $CDCl_3$): 7.21-6.99 (m, 24H), 4.30 (s, 34H), 3.77 (s, 25H), 3.58 (s, 190H), 3.20 (s, 25H), 2.84 (s, 25H), 2.62 (s, 25H), 1.95-1.66 (m, 108H), 1.44 (s, 18H), 1.36-0.73 (m, 527H)

GPC analysis: $M_w$ = 5387 $gmol^{-1}$, $M_n$ = 3065 $gmol^{-1}$, PD = 1.76. DSC Tg = 53.76 °C. TGA decomposition at 213 to 501 °C.

### 1.6.7 Large scale DPA protected functional polymers

*Table S8: Monomer and initiator weight and mole percent values used to formulate large scale DPA functional polymers.*

| | Weight percent | | | | | Mol % | |
|---|---|---|---|---|---|---|---|
| Polymer | MMA | GMA | STY | IBOMA | DPA | Radical int | Total mass (g) |
| 5-DPA-HT* | 0.47 | 0.28 | 0.04 | 0.16 | 0.05 | (T) 0.04 | 815 |

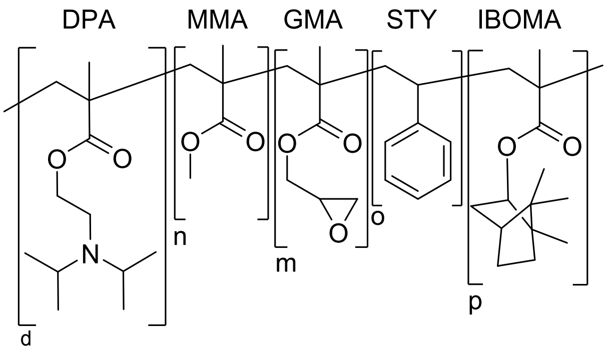


*Figure S5: General structure of the large-scale DPA functional polymer.*

5-DPA-HT*

$\delta_H$ (400 MHz; $CDCl_3$): 7.20-7.00 (m, 24H), 4.30 (s, 34H), 3.77 (s, 30H), 3.58 (s, 182H), 3.21 (s, 25H), 2.84 (s, 33H), 2.62 (s, 25H), 2.14-0.53 (m, 669H)

GPC analysis: $M_w$ = 18138 $gmol^{-1}$, $M_n$ = 2281 $gmol^{-1}$, PD = 7.95. DSC Tg = 55.05 °C. TGA decomposition at 219 to 499 °C.

## 1.7 $T_g$ (DSC) of polymers

a) CTL-HT* (LS)

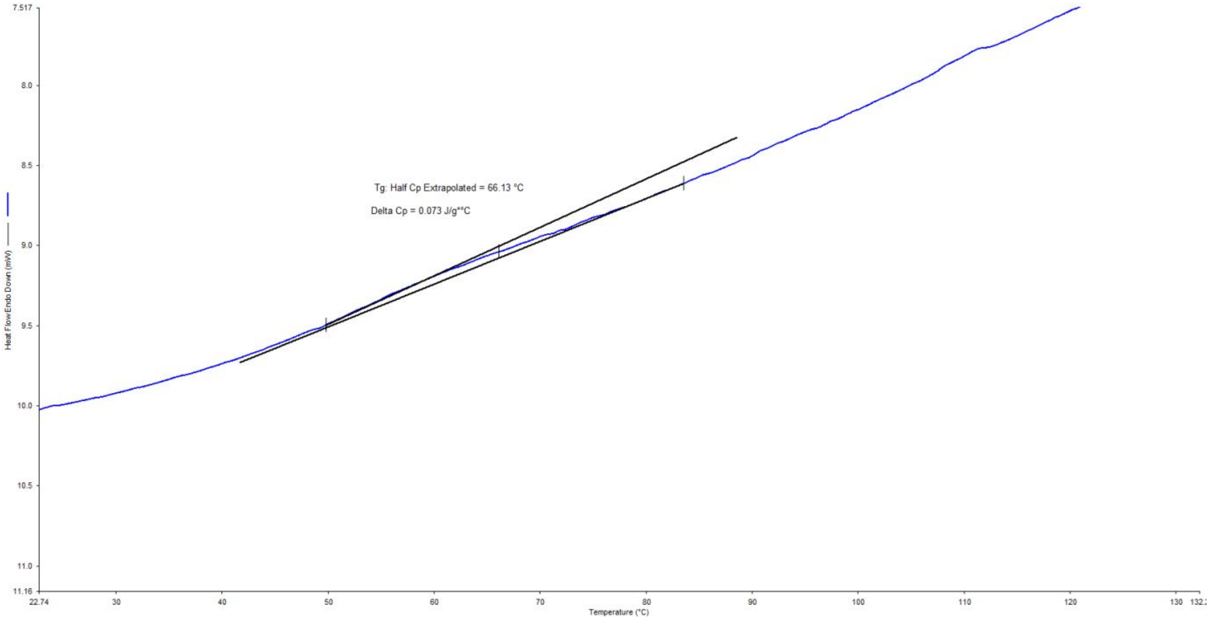


b) CTL- LT

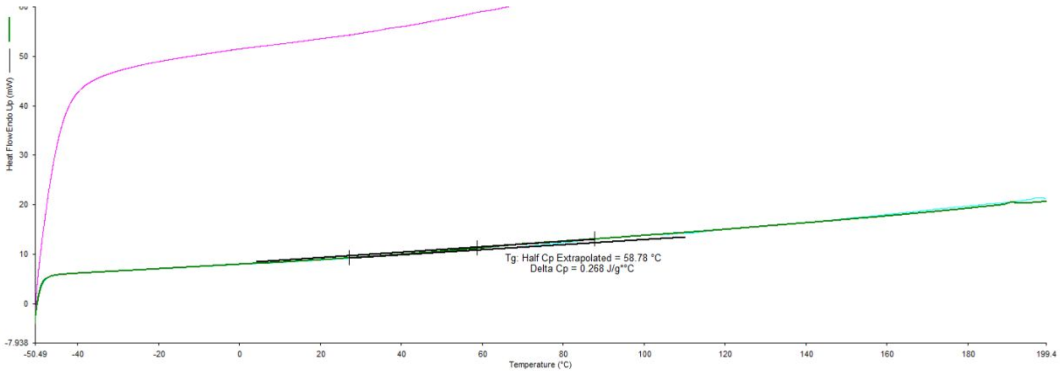

c) 5-BOC-HT

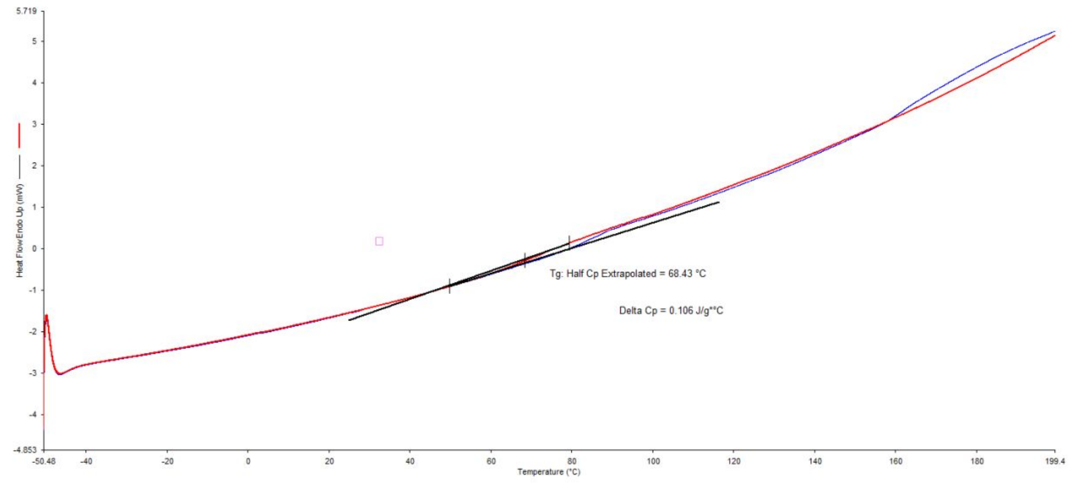


d) 5-BOC-LT

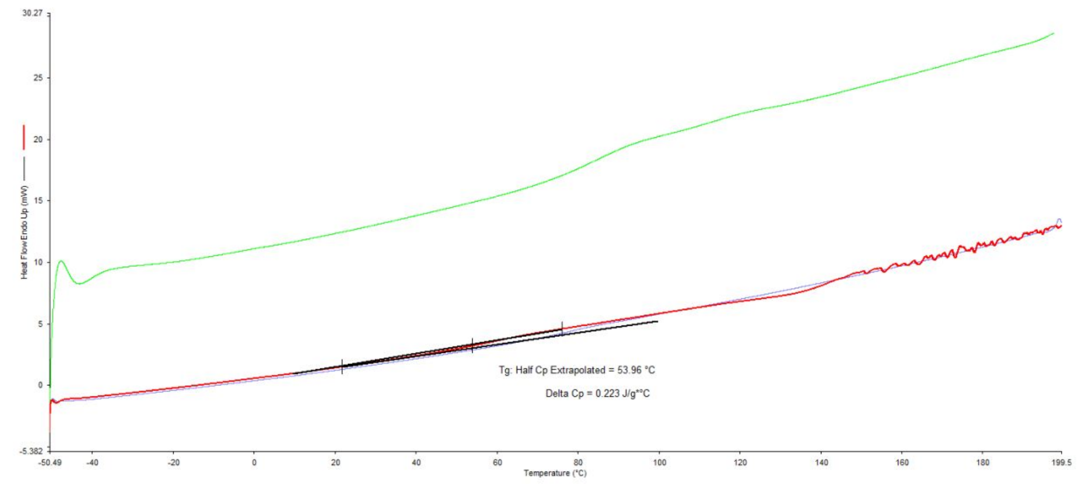

e) CTL-HT* (LS)

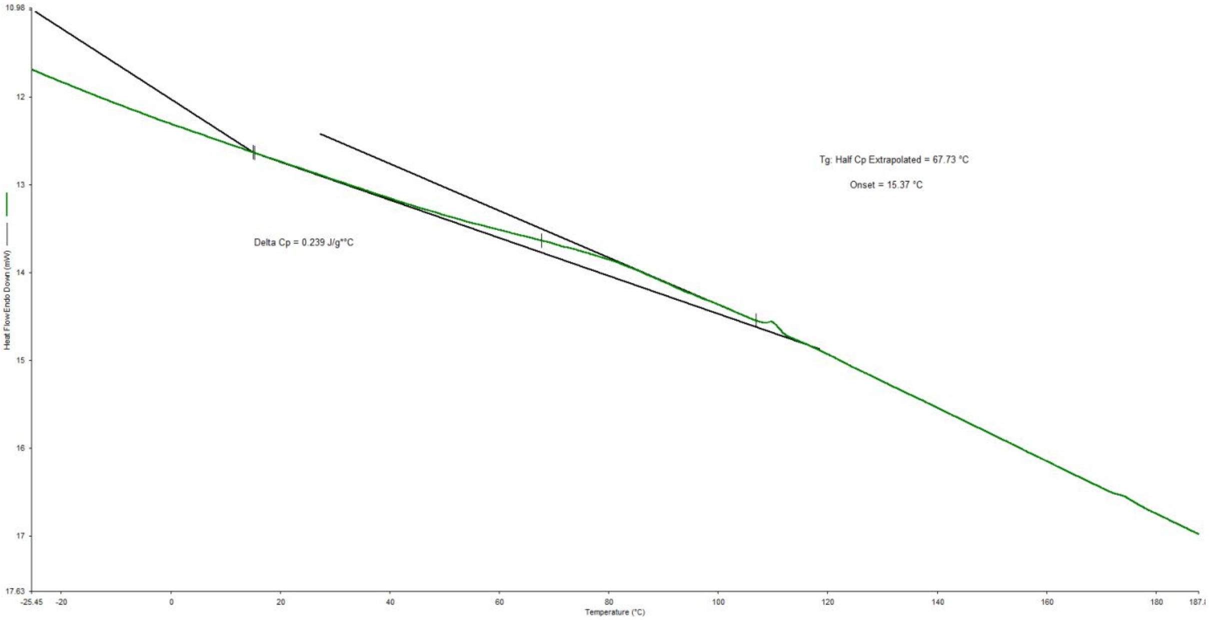


f) 5-BOC-HT* (LS)

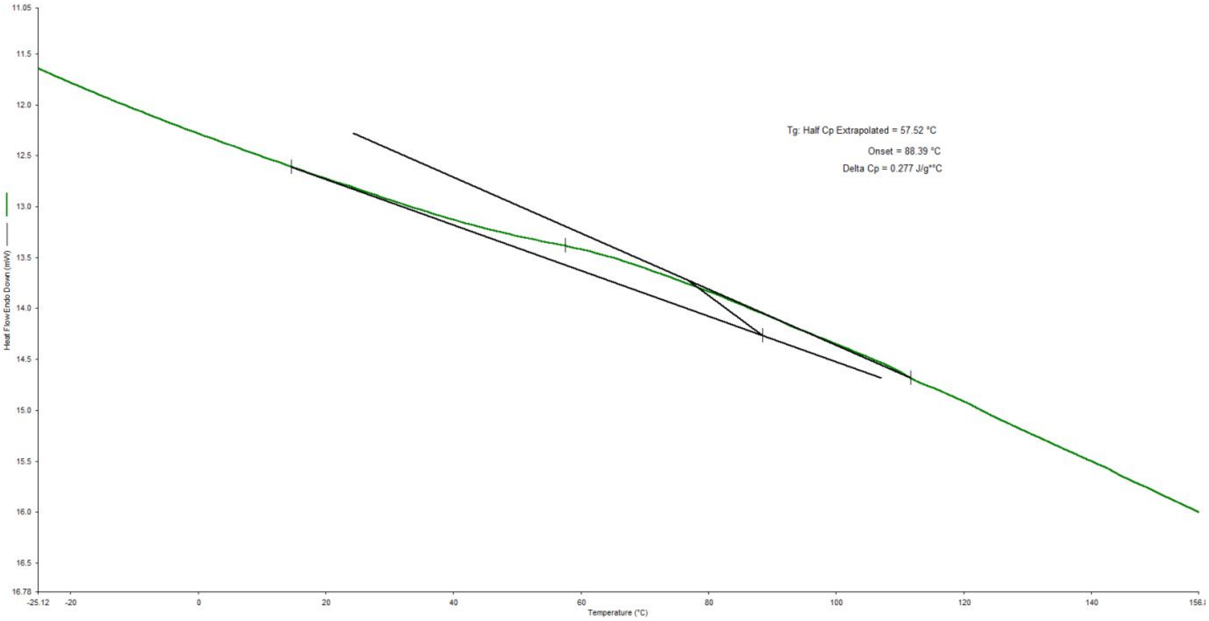

g) 5-BOC-LT*

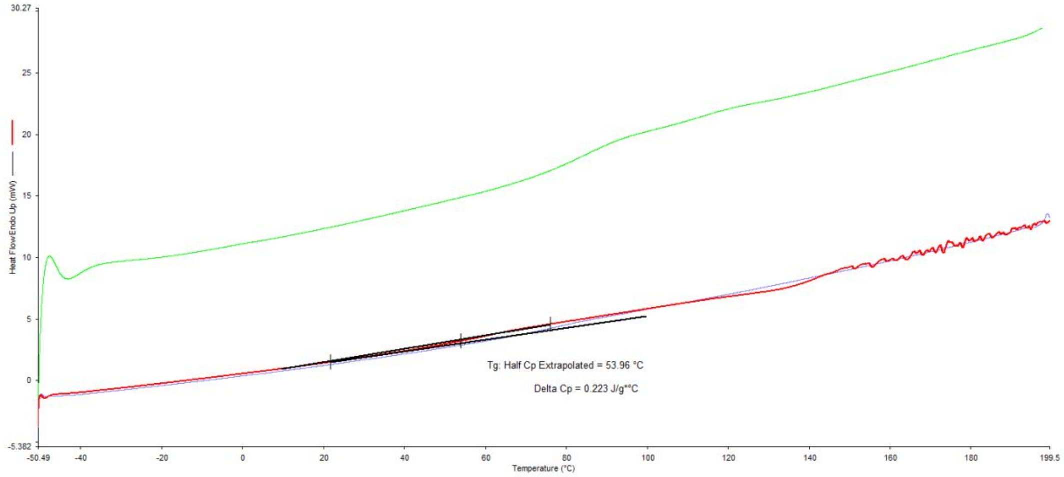


h) 5-DPA-HT* (LS)

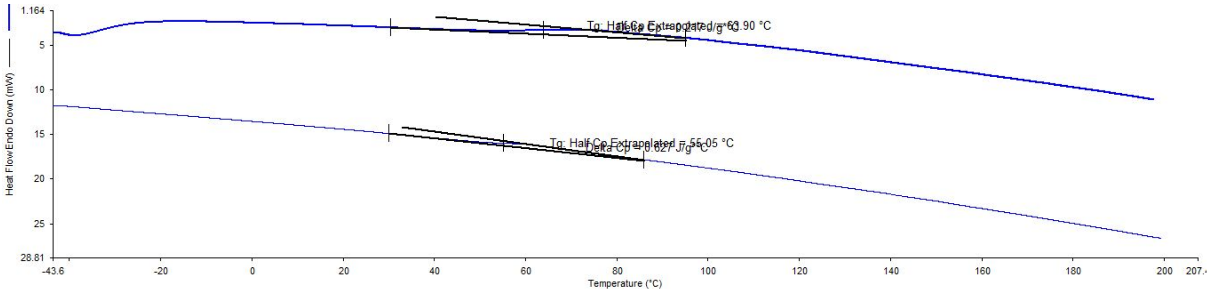


*Figure S6 a-h): DSC traces for polymers used in this study and used to provide $T_g$ data presented in main manuscript.*

## 1.8 NMR spectra images

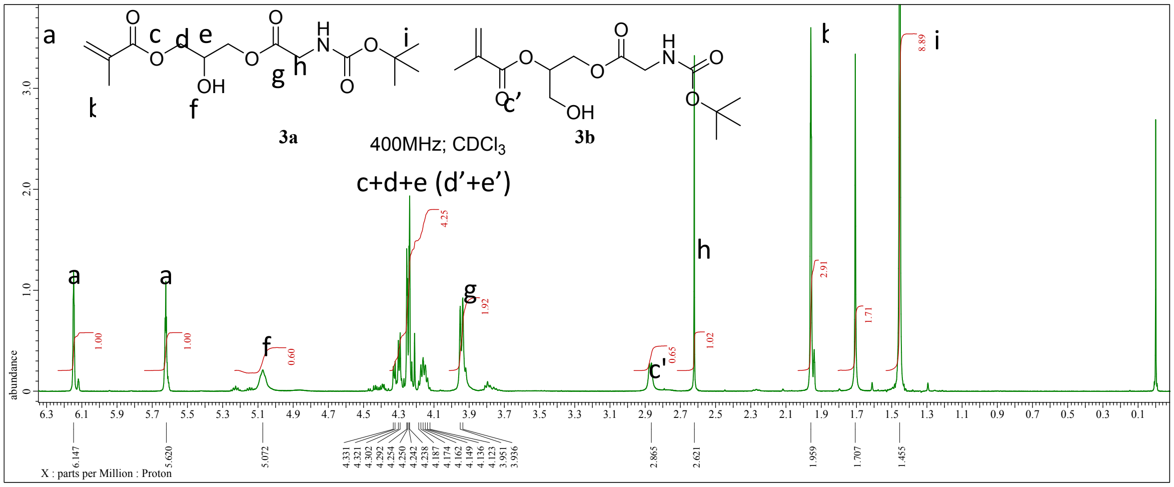

CTL-HT

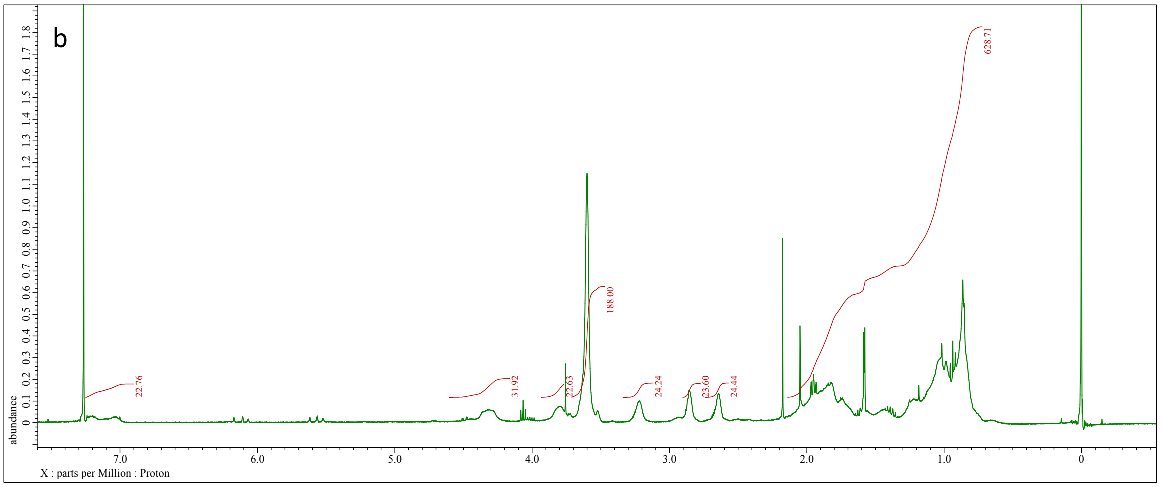
b
abundance
X : parts per Million : Proton
7.0
6.0
5.0
4.0
3.0
2.0
1.0
0
22.76
31.92
22.63
188.00
24.24
23.60
24.44
628.71

CTL-LT

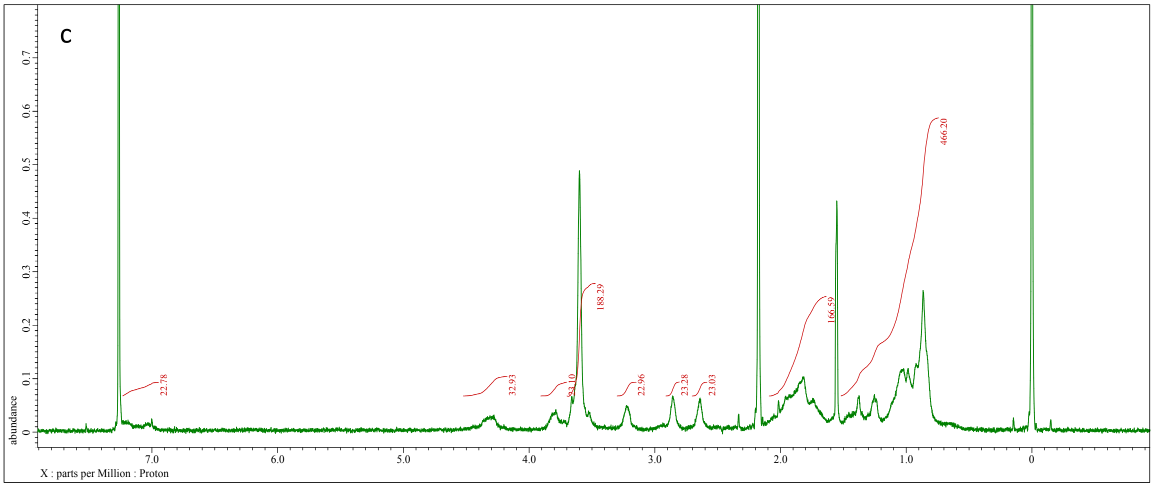
c
abundance
X : parts per Million : Proton
7.0
6.0
5.0
4.0
3.0
2.0
1.0
0
22.78
32.93
188.29
22.96
23.28
23.03
166.59
466.20

50-BOC-HT

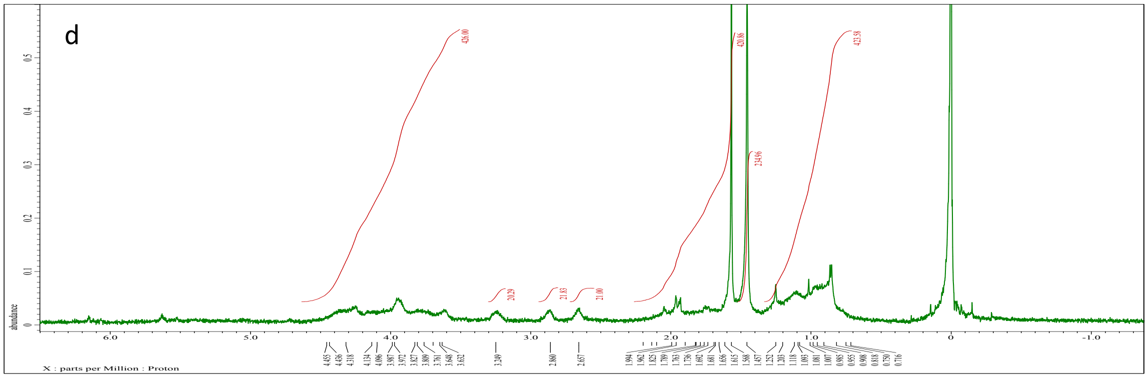


5-BOC-HT

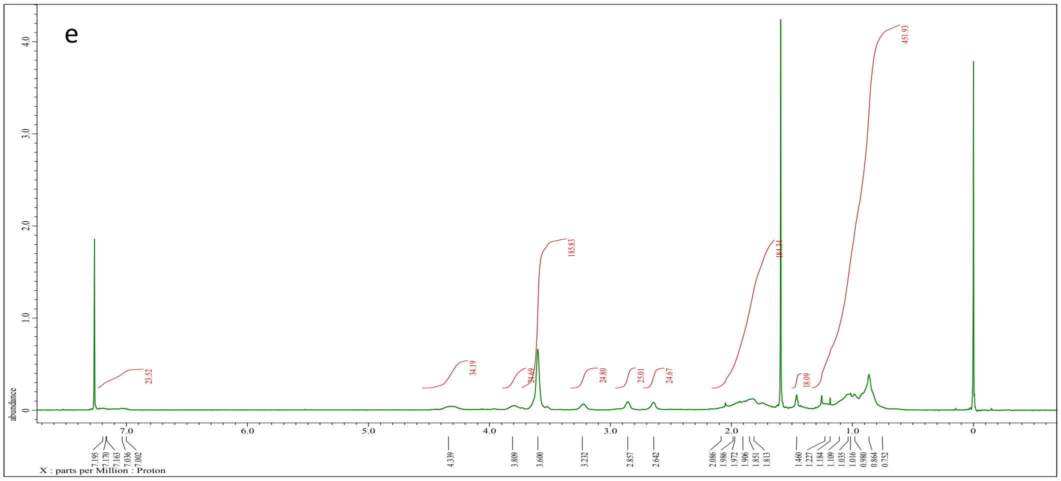


5-BOC-LT

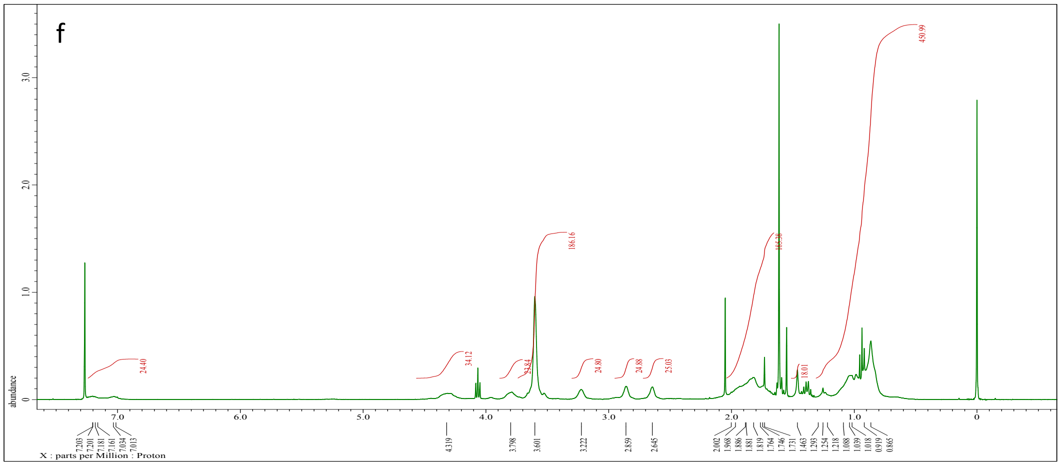

CTL-HT*

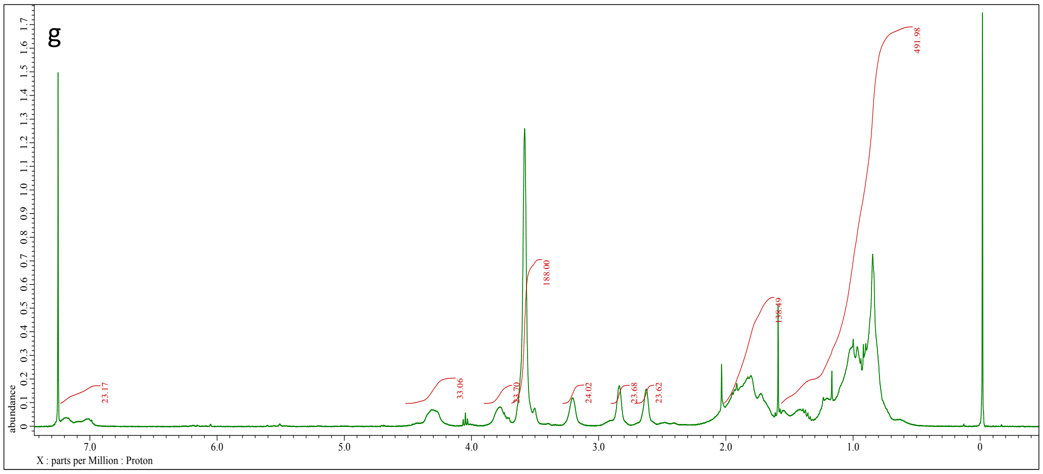


5-BOC-HT*

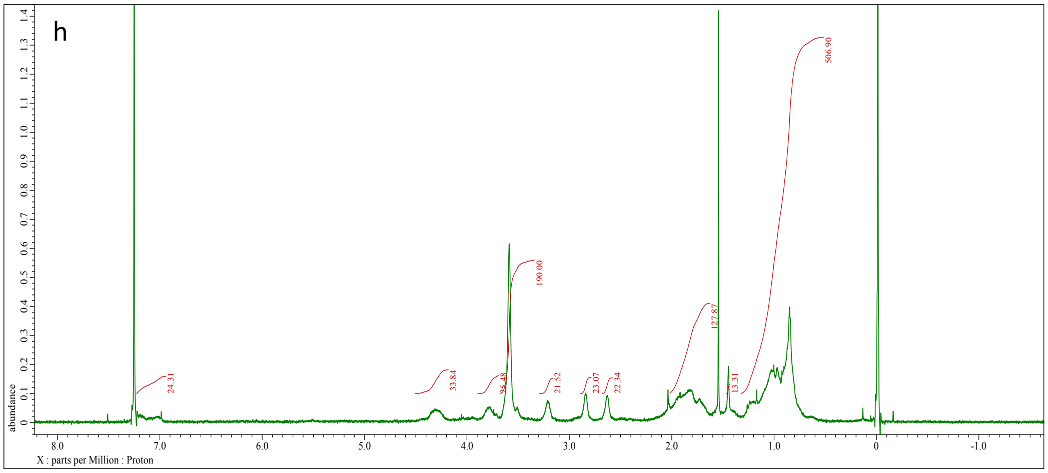

5-BOC-LT*

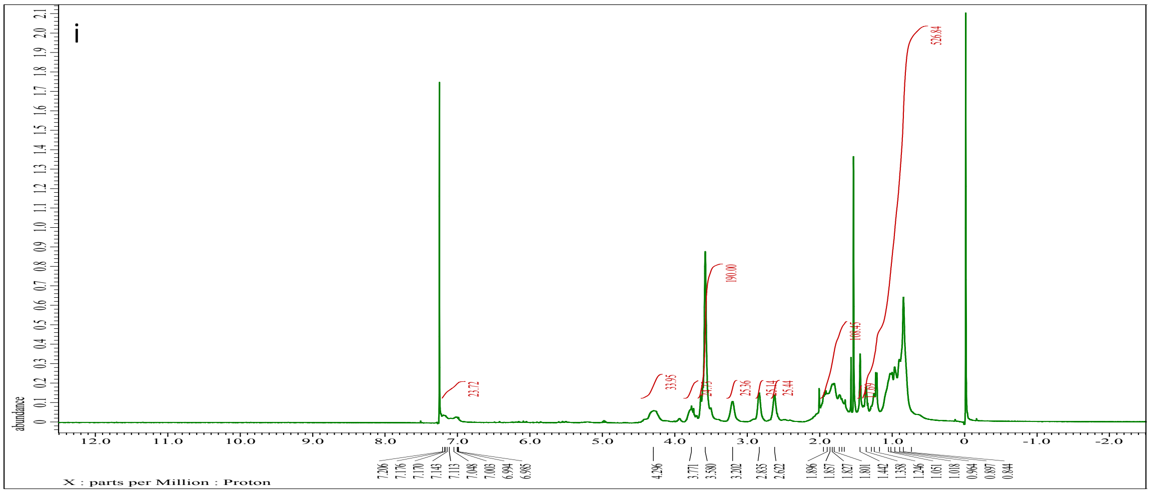


5-DPA-HT*

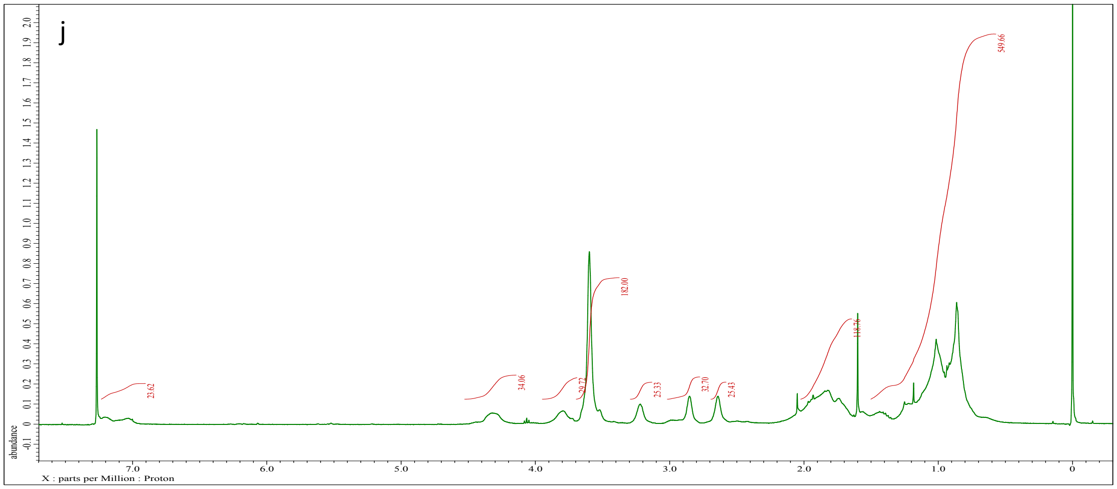


*Figure S7 a-j: NMR spectra of BOC-Gly-MA (a) and polymers (b-j) used in this study.*

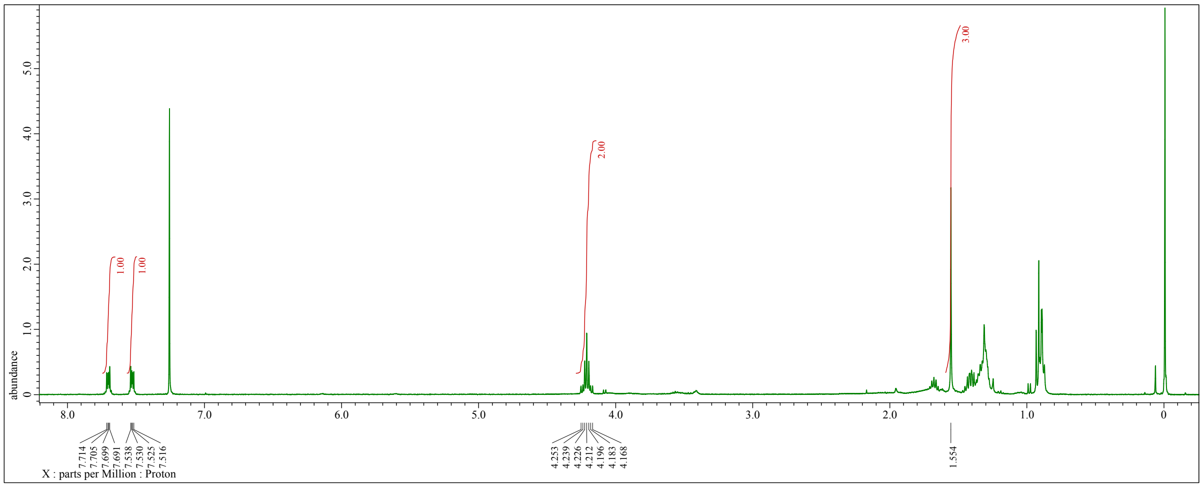


*Figure S8: $^{1}H$-NMR (crude) of the mixed aromatic (colored) impurity from the BOC-Gly-MA synthesis, when using tert-butylhydroquinone as a radical scavenger to suppress polymerisation.*

## 1.9 Analytical experiments and calculations

The integration of *the* BOC-NHC$\boldsymbol{H_2}$-R (B) against the unprotected $NH_2$-C$\mathbf{H_2}$-R (A) allowed for an estimation of the degree of BOC protection group removal when heated using **equation S1** below.

*Table S9: Results presenting relative peak intensities for both BOC-LT and BOC-HT samples, with the calculated loss in mass presented in the final column.*

| | **TGA analysis** | | | **$^{1}$H-NMR analysis** | | |
|---|---|---|---|---|---|---|
| **Sample** | **A** | **B** | **BOC loss %** | **R-$NCH_2$ Peak integration** | **BOC-$NCH_2$ Peak integration** | **BOC Loss (%)** |
| BOC-LT | 0.028 | 1.99 | 1.4 | 0.028 | 1.99 | 1.4 |
| BOC-HT | 4.62 | 1.97 | 69.8 | 4.62 | 1.97 | 69.8 |

**S1**

$$\frac{A}{(A+B)} * 100 = BOC\ loss\ \%$$

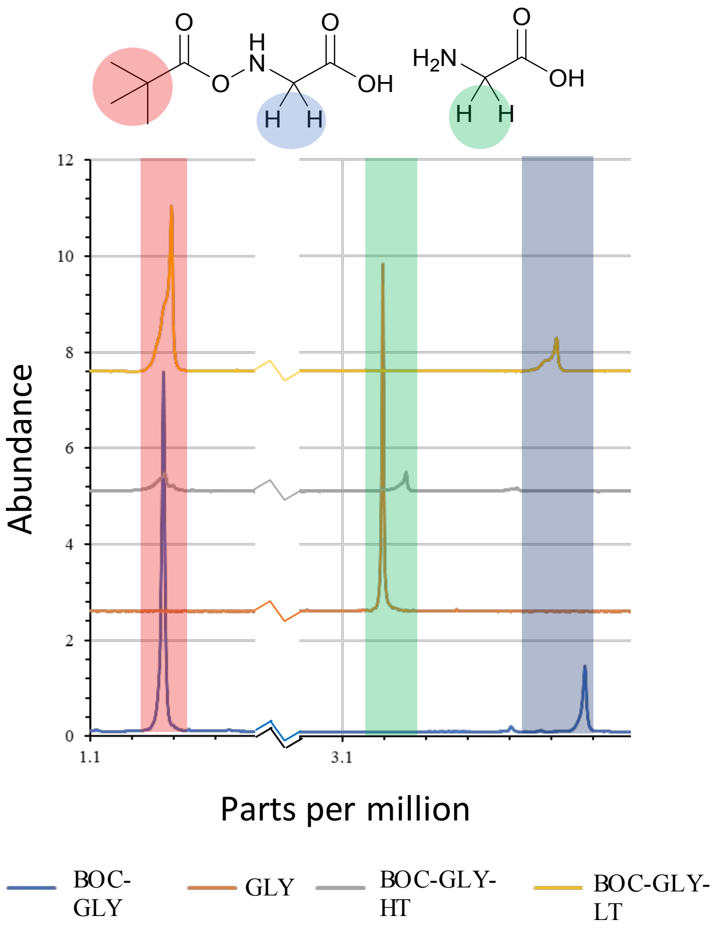


*Figure S9: ¹H-NMR Spectra analyzed to determine the percentage of BOC groups remaining after TGA experimentation.*

## 1.10 Model acidic corrosion study

Functional co-polymer (500 mg, 50-BOC-HT) was placed in a glass vial containing 8mL of 4M HCl(aq)/THF (1:1 v/v). IR (Bruker alpha Platinum-ATR, 32 scans, 4000 to 650 $cm^{-1}$) scans were performed on extracted polymer samples once every 24h for a total of 7 days. To obtain each polymer sample a 0.5 mL aliquot of the reaction solution was diluted in 5mL of DCM, this solution was then washed with 5mL of saturated

$NaHCO_{3(aq)}$. The organic phase was collected and dried over $MgSO_4$, solvent was subsequently removed under reduced pressure, to afford a dried polymer sample for IR analysis. $^1$H-NMR analysis was also performed on a sample taken prior to emersion in the aqueous solution, and one on the 7th day extraction in the aqueous solution, the spectra are shown in Figure S10.

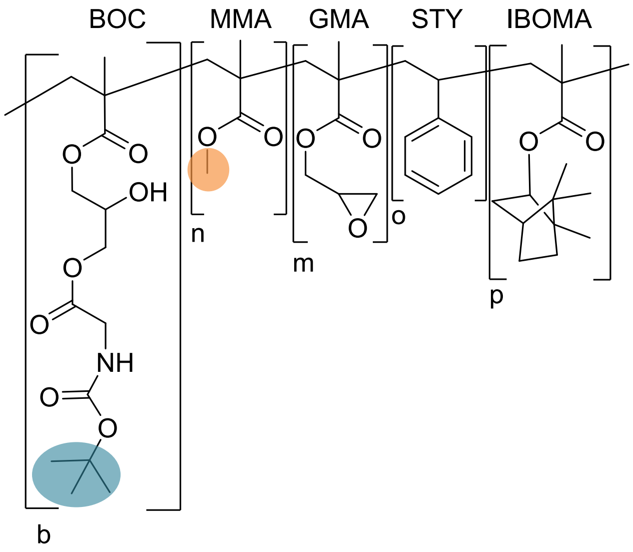

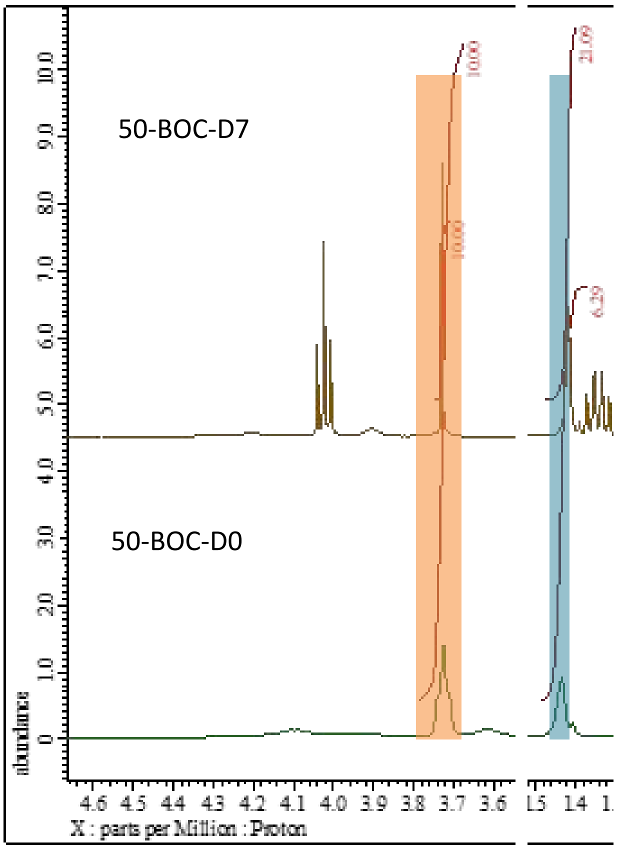


*Figure S10: $^{1}$H-NMR Spectra analyzed to determine the percentage of BOC groups remaining after model corrosion study.*

## 1.11 BOC functionality at the coating-metal interface study

To directly measure the impact of filiform corrosion on a functional thermoset coating, a new experimental method was developed. It was designed to allow for direct access to the coating aluminum interface without the requirement for destructive techniques (i.e., removal of coating from the aluminum substrate by scalpel). High weight percent (50% w/w) polymers capable of curing (50-BOC-HT) were synthesized to provide increased ease of detection of functional group transformation. The polymer coating was also adhered to a separate aluminum substrate to maintain structural integrity during the removal of corrosion products.

50-BOC-HT solutions were formulated utilizing the same additive composition to that of their powder coating (Table S2) but applied as a solution (liquid) paint in *n*-butyl acetate. Aeroxide Alu C was excluded from the coating solution formulation, as its role as an anticaking agent is not required in the absence of powder particulate. Solution application required 8 g of 50-BOC-HT polymer to coat the area of aluminum substrate required for studying changes in the concentration of BOC functionality at the coating-metal interface.

Solution paints used to produce highly functional coatings were manufactured by first forming a solution of 8 g of the polymer (50-BOC-HT) in *n*-butyl acetate (10 mL) heated to 60°C, before the addition of Resiflow PL-200 (1 mL, 0.1 g/mL in *n*-butyl acetate) followed by 0.1 g of benzoin. Powdered (50 µm) sebacic acid (1.6 g, cross-linker) was then added to the solution and vigorously stirred to produce a uniform suspension.

Before application, the aluminum substrate was cleaned with deionized water followed by industrial methylate spirits (IMS), the aluminum substrate was then dried at room temperature. Tape was then adhered across the edge of the aluminum substrate to both smooth the movement of a draw-down bar and contain the polymer solution within the center of the panel. The paint solution was then applied to the aluminum substrate using a 400 µm drawdown bar, *n*-butyl acetate was removed from the coated aluminum substrate by drying at room temperature for 48 hours. Once dry, the tape was removed and the coating was cured at 200 °C for 5 minutes followed by a 25-minute post cure at 185 °C, in-line with a standard powder coating cure schedule.

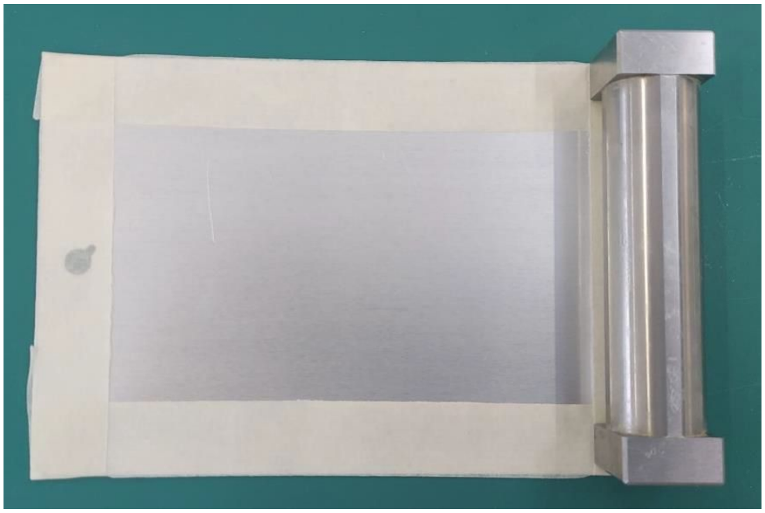

*Figure S11: Drawdown bar and taped aluminum substrate before coating application.*

The coated aluminum panel was cut into 30 x 30 mm squares, which were then placed in-between a square of PTFE and a secondary thicker piece of cast aluminum (aluminum substrate B, see Figure S12), both cut to 35 x 35 mm. The polymer coated surface of the substrate samples was placed in contact with the cast aluminum substrate B, as illustrated below. The difference in area between the coated substrate (30 x 30 mm) and the aluminum substrate B (35 x 35 mm) was employed to produce an outer edge of uncovered aluminum which was susceptible to corrosive attack. As such filiform corrosion would initiate from the exposed edge, and propagate across the aluminum substrate B surface, whilst interacting with the coated substrate secured on top, simulating the formation of filiform corrosion in a coated product which has experienced damage.

The current study focuses on the interaction between the aluminum substrate B and the functional coating surface, and not the relationship between the functional coating and the aluminum (Q) panel. As such, the variation in coating thickness resulting from the addition of the tape was not theorized to have a significant effect on the results of the study.

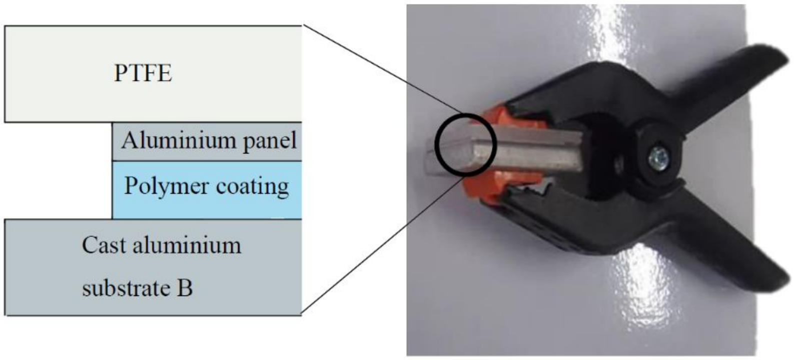


*Figure S12: Assembled clamped sample of 50-BOC-HT prior to accelerated corrosion testing.*

## 1.12 Filiform corrosion test procedure (SAE J2635)

Quantification of filiform corrosion (FFC) present on all coated aluminum substrates followed the process set out by the SAE (society of automotive engineers) J2635 filiform corrosion test procedure for painted aluminum wheels and painted aluminum wheel trim. To initiate the accelerated corrosion process, each coated aluminum substrate was scribed to remove the protective polymer coating and expose the underlying aluminum substrate using a carbide tipped blade, for a length of 100 mm using a guide to reliably produce a straight line. Scribed samples are then placed into a copper accelerated salt spray (CASS) chamber at a 45° angle for 6 hours, upon completion the samples are washed with deionized water.

The coated aluminum substrates are then placed into a humidity chamber (60±1 °C, 85% relative humidity ±3%) for a period of 30 days, at an angle of 45° to allow for moisture run off. FFC length measurements are recorded perpendicular to the scribe, without including the scribe. FFC number was recorded from

both sides of the scribe omitting the first and final 5 mm of the scribe. FFC length and number were recorded both manually, and automatically using Quantiz measurement techniques. Whereas corroded surface area and average FFC width measurements were only obtainable using Quantiz analysis.

$$\frac{(\Sigma_{SA}/\Sigma_{NO})}{A_L} = FFC_w \quad \textbf{S2}$$

**Equation S2** was used to calculate the average FFC width ($FFC_w$) present on an aluminum substrate, post exposure to accelerated corrosion conditions. Where $\Sigma_{SA}$ is the total corroded surface area, $\Sigma_{NO}$is the total number of FFC and $A_L$is the average length of a single FFC filament, where each measurement ($\Sigma_{SA}$, $\Sigma_{NO}$, $A_L$) is recorded for every sample panel in a coating series.

## 1.13 Statistical testing data

Analysis was conducted using median absolute deviation (MAD) for comparison of FFC length, as the results were not normally distributed. MAD was used as it is a more robust measurement of non-normally distributed data, compared to standard deviation (SD). The non-normal distribution is a result of the relatively low presence of longer FFC trails compared to a higher abundance of shorter trails, causing a tailing effect.

Standard deviation (SD) was used rather than MAD when plotting FFC number and CSA as the results were normally distributed. Due to the smaller data series produced by FFC Number and FFC CSA (4

rather than >200) statistical analysis was not performed due to the potential to encounter type 2 error.

All statistical results used to plot results is recorded in Table S10 to Table S13.

Table S10: Results produced from corrosion data which was processed through JASP software.

| Sample | **FFC length** | MAD | **FFC number** | SD | **FFC CSA** | SD |
|---|---|---|---|---|---|---|
| **CTL** | 1.07 | 0.581 | 60.75 | 4.79 | 118.54 | 82.89 |
| **COM** | 1.006 | 0.452 | 60.75 | 2.63 | 63.37 | 26.53 |
| **5-BOC-LT** | 0.748 | 0.388 | 50.75 | 3.5 | 52.69 | 20.25 |
| **5-BOC-HT** | 1.619 | 0.549 | 64 | 8.9 | 127.3 | 18.51 |
| **5-DPA-HT** | 1.329 | 0.581 | 61 | 10.55 | 90.47 | 26.35 |

*Table S11: Results of ANOVA testing.*

**ANOVA – FFC length (mm)**

| Cases | Sum of Squares | df | Mean Square | F | p |
|---|---|---|---|---|---|
| Quantiz Sample | 117.359 | 4 | 29.340 | 22.192 | < .001 |
| Residuals | 1565.377 | 1184 | 1.322 | | |

*Note.* Type III Sum of Squares

*Table S12: Results of Dunn's Post Hoc comparison testing.*

**Dunn's Post Hoc Comparisons – FFC length**

| Comparison | z | $W_i$ | $W_j$ | p | |
|---|---|---|---|---|---|
| 5-BOC-HT - 5-BOC-LT | 10.179 | 768.521 | 440.116 | < .001 | *** |
| 5-BOC-HT - 5-DPA-HT | 4.625 | 768.521 | 626.469 | < .001 | *** |
| 5-BOC-HT - COM | 8.728 | 768.521 | 500.177 | < .001 | *** |
| 5-BOC-HT - CTL | 5.325 | 768.521 | 604.809 | < .001 | *** |
| 5-BOC-LT - 5-DPA-HT | -5.714 | 440.116 | 626.469 | < .001 | *** |
| 5-BOC-LT - COM | -1.840 | 440.116 | 500.177 | 0.033 | * |

**Dunn's Post Hoc Comparisons – FFC length**

| Comparison | z | $W_i$ | $W_j$ | p | |
|---|---|---|---|---|---|
| 5-BOC-LT - CTL | -5.045 | 440.116 | 604.809 | < .001 | *** |
| 5-DPA-HT - COM | 4.059 | 626.469 | 500.177 | < .001 | *** |
| 5-DPA-HT - CTL | 0.696 | 626.469 | 604.809 | 0.243 | |
| COM - CTL | -3.360 | 500.177 | 604.809 | < .001 | *** |

* p < .05, ** p < .01, *** p < .001

*Table S13: Results of Kruskal-wallis testing*

**Kruskal-Wallis Test**

| Factor | Statistic | df | p |
|---|---|---|---|
| Quantiz Sample | 127.519 | 4 | < .001 |
